# Hydrogen‐Free APCVD Synthesis of Heterophase WSe_2_ Nano‐Butterflies for Room Temperature NO_2_ Detection: Experimental and Computational Insights

**DOI:** 10.1002/smsc.70322

**Published:** 2026-06-08

**Authors:** Mubdiul Islam Rizu, Abhilash Patra, Fatima Ezahra Annanouch, Milica Todorović, Dalal Fadil, Eduard Llobet

**Affiliations:** ^1^ Universitat Rovira i Virgili MINOS School of Engineering Tarragona Spain; ^2^ IU‐RESCAT Research Institute in Sustainability Climatic Change and Energy Transition Universitat Rovira i Virgili Vila‐seca Spain; ^3^ TecnATox ‐ Centre for Environmental Food and Toxicological Technology Universitat Rovira i Virgili Tarragona Spain; ^4^ Department of Mechanical and Materials Engineering Faculty of Technology University of Turku Turku Finland

**Keywords:** 2D materials, atmospheric pressure chemical vapor deposition, density functional theory, gas sensor, heterophase, NO_2_, transition metal dichalcogenides

## Abstract

We report a scalable hydrogen‐free synthesis of heterophase 2H/1T′ WSe_2_‐based gas sensor for ultrasensitive room temperature NO_2_ detection. Moving beyond conventional defect engineering, this work leverages deliberate phase and morphological synergies to overcome traditional kinetic trade‐offs in 2D material‐based sensors. Unlike conventional 3D structures, this open hierarchical nano‐butterfly‐like morphology prevents van der Waals restacking and maximizes exposed active edge sites for rapid gas diffusion. Quantitative phase engineering yielding an optimized surface of kinetically trapped 1T′ domains (∼17%) within a dominant 2H matrix (∼83%) uniquely couples the semiconducting baseline stability of the 2H phase with the rapid, metallic charge–transfer channels of the 1T′ twin boundaries. This synergistic transduction mechanism demonstrated a remarkable 40% response toward 800 ppb NO_2_, a theoretical limit of detection (LOD) of 4 ppb, and robust environmental stability over 40 weeks without requiring external thermal or optical activation. Furthermore, density functional theory (DFT) simulations accelerated by Bayesian optimization successfully validated the experimental findings, identifying physisorption as the dominant interaction mode for NO_2_ on WSe_2_ and providing insight into different adsorption modes on 2H and 1T′ domains. These results establish a highly quantitative, structurally engineered framework for next‐generation low‐power transition metal dichalcogenides (TMDs) based gas sensors.

## Graphical abstract

Scalable, hydrogen‐free atmospheric pressure chemical vapor deposition synthesis of heterophase 2H/1T′ WSe_2_ for ultrasensitive room‐temperature NO_2_ detection. Hierarchical nano‐butterfly morphology prevents restacking and maximizes active edges. Optimized phase engineering (~17% 1T′) enables fast charge transfer, achieving 40% response at 800 ppb, 4 ppb limit of detection, long‐term stability, and density functional theory‐validated physisorption‐driven sensing.

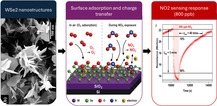

## 1 Introduction

Global initiatives for energy sustainability are intrinsically linked to the mitigation of nitrogen dioxide (NO_2_) emissions through the accelerated transition toward renewable energy sources [1, 2]. The anthropogenic combustion‐induced reactive nitrogen species adversely affect air quality, ecosystem integrity, and global climate through numerous pathways [3]. NO_2_ pollution arising from the excessive reliance on conventional fossil energy reserves impedes the realization of the clean energy goals (SDG 7) envisioned by the United Nations (UN) [4]. According to the legal framework proposed by the Scientific Committee on Occupational Exposure Limits (SCOEL) of the European Commission, the permissible 8 h time‐weighted average (TWA) exposure to NO_2_ is restricted to 0.5 ppm, while the short‐term exposure limit (STEL) for a 15 min interval is set at 1 ppm [5]. Regular inhalation of NO_2_ can increase the risk of chronic respiratory infections, asthma exacerbations, and cardiovascular morbidity [6, 7]. Moreover, excessive exposure can induce severe respiratory dysfunction leading to the paralysis of the pulmonary system [8]. Therefore, precise detection of trace level NO_2_ is required for effective air quality monitoring, which complements broader efforts toward energy sustainability, public health safety, and environmental conservation.

In the contemporary landscape of environmental monitoring, semiconductor‐based chemiresistive gas sensors have been extensively used in a broad range of gas sensing applications, achieving remarkable commercial success. Their widespread deployment is attributed to a simple yet effective transduction mechanism, scalable fabrication, low cost, suitability for miniaturization, and real‐time monitoring capacity [9]. These sensors typically incorporate an insulating substrate, interdigitated electrodes, and a microheater. Their operating principle is governed by the modulation of free charge carrier concentration within the gas‐sensitive layer upon exposure to target gaseous analytes. In this aspect, metal oxide semiconductors (MOXs) have been used as sensing materials over the years [10]. To date, a wide array of MOX nanostructures exhibiting one‐dimensional (1D) (nanorods, nanowires, nanoribbons), two‐dimensional (2D) (nanosheets, nanoplates), and three‐dimensional (3D) (nanoarrays, nanoflowers) morphologies have already been explored [11]. Despite their advantages like robustness, electron confinement effect, low defects, high sensitivity, and low cost, MOX‐based gas sensors endure poor selectivity, prolonged recovery time, and baseline drift. Nonetheless, the requirement of high operating temperature (typically 100°C to 400°C) remains one of the major drawbacks for using them in IoT‐compatible gas monitoring networks [12].

In search of viable alternatives to MOX, a range of low‐dimensional material‐based gas sensors have been investigated. Among them, nanostructured conductive polymers have emerged as compelling materials owing to their low temperature applications, ease of fabrication, and tunability [13]. Also, carbon nanotubes (CNTs), having a distinctive porous structure, exhibit high sensitivity and mechanical flexibility [14]. However, these materials are also affected by humidity interference, slow recovery kinetics, material degradation, and complex manufacturing process. The discovery of graphene and similar 2D materials has brought a revolutionary change in the field of gas sensing [15, 16]. Researchers have been exclusively examining graphene due to its superior carrier mobility, the possibility of functionalization, and large surface area. Nevertheless, its zero bandgap and lack of environmental durability remain key challenges for practical deployment. Moreover, the incorporation of any non‐carbon elements during synthesis can induce structural defects affecting its gas sensing performance [17].

Transition metal dichalcogenides (TMDs) have been widely investigated as promising candidates for next‐generation IoT‐enabled gas sensing platforms [18]. These remarkable 2D van der Waals (vdW) materials exhibit a unique combination of physicochemical and electronic properties, such as layer‐dependent bandgap, high on/off current ratio, strong light matter interaction, high charge carrier mobilities, large surface to volume ratio, tunable chemical properties, strong spin–orbit coupling, and optimum surface energy for target gas adsorption [19]. A wide range of TMDs based gas sensors have already shown promising sensitivity during room temperature operation [20]. In particular, layered tungsten diselenide (WSe_2_) has a growing prospect for selective NO_2_ sensing, due to its expansive surface area and tailored bandgap [21]. Furthermore, a theoretical study employing first‐principles computations has also revealed strong chemisorption of NO_2_ over the WSe_2_ sensing layer [22].

To date, WSe_2_ has been synthesized via liquid‐phase exfoliation (LPE) [23, 24], hydrothermal synthesis [25], and chemical vapor deposition (CVD) [26, 27]. While solution‐processed and hydrothermal approaches are accessible, they inherently suffer from residual surfactants, poor inter‐flake electrical contact, and uncontrolled defect densities [28]. These wet‐chemistry limitations often translate to severe baseline drift and poor device integration in gas sensors [29]. Conversely, CVD offers rapid, direct‐to‐substrate growth of edge‐enriched 3D films with superior electrical integrity. However, conventional CVD‐grown 3D WSe_2_ structures, such as randomly agglomerated nanoflowers, often suffer from dense core packing that traps analyte molecules, severely hindering recovery kinetics [30]. Furthermore, most CVD approaches rely heavily on hydrogen assistance to reduce metal oxide precursors [31]. At elevated temperatures, hydrogen reacts with selenium and forms hydrogen selenide (H_2_Se), which is a highly volatile and lethal environmental hazard [32, 33, 34]. Furthermore, H_2_‐assisted selenization requires a specialized CVD configuration equipped with flashback arrestors and real‐time gas monitoring to ensure concentrations remain well below the lower explosive limit (LEL).

To overcome these critical bottlenecks, there is an urgent need to move beyond routine morphological variations and engineer specifically tailored, scalable architectures. Recent high‐quality reports on TMD‐based NO_2_ sensors have highlighted the critical need to balance defect engineering, phase composition, and hierarchical structure to achieve room‐temperature operation without sacrificing recovery time or environmental robustness [35, 36, 37]. On the other hand, concurrent work on molybdenum‐based gas sensors revealed that chemical vapor reactions of the precursors in a hydrogen‐free environment can significantly improve the overall gas sensing performance [38]. Addressing this precise gap, we report a highly scalable, hydrogen‐free atmospheric pressure CVD (APCVD) assisted synthesis of heterophase (2H/1T′) WSe_2_ “nano‐butterflies”. This methodology ensures direct integration onto sensor substrates, circumventing the toxicity of traditional CVD while avoiding the inter‐flake resistance of wet chemistry. The unique open‐channel morphology prevents structural restacking and preserves active surface area. Simultaneously, the engineered coexistence of the 2H and 1T′ phases synergizes the semiconducting stability of the 2H phase with the highly active, metallic charge–transfer channels of the 1T′ domains, drastically lowering the activation energy for NO_2_ adsorption at room temperature.

In this study, we have developed a hydrogen‐free simple, and rapid synthesis method by employing APCVD. The WSe_2_ thin films were directly synthesized on SiO_2_/Si substrate without using any catalyst seeding or external assistance (e.g., pressure, plasma, etc.). The as‐grown film morphology was thoroughly investigated by field emission scanning electron microscopy (FESEM) and transmission electron microscopy (TEM). The elemental analysis was performed by energy‐dispersive X‐ray spectroscopy (EDX). The lattice structure was verified by X‐ray diffraction (XRD). High‐resolution transmission electron microscopy (HRTEM) and corresponding fast Fourier transform (FFT), as well as selected area electron diffraction (SAED) were used for in‐depth investigation of crystallinity and lattice phase. The molecular fingerprint and the chemical composition analysis were conducted by Raman spectroscopy and X‐ray photoelectron spectroscopy (XPS), respectively. Later on, the sensors were tested against low concentrations of NO_2_ gas at room temperature. The cross‐sensitivity and the impact of ambient moisture on sensing performance were also rigorously assessed. To elucidate the atomistic interaction mechanism between NO_2_ and the sensing substrate, we performed density functional theory (DFT) calculations. The identification of the energetically favorable adsorption configuration was streamlined via Bayesian optimization, enabling an accelerated exploration of the potential energy surface. For the most stable adsorption geometries, we proceeded with a detailed analysis of the electronic structure properties to investigate the nature of the gas‐surface bonding mechanisms, charge transfer, and electronic level alignment. In the end, a comprehensive gas sensing mechanism was proposed by reconciling our experimental observations with the electronic level insights derived from DFT calculations.

## 2 Experimental Details

### 2.1 Material Synthesis

**FIGURE 1:**
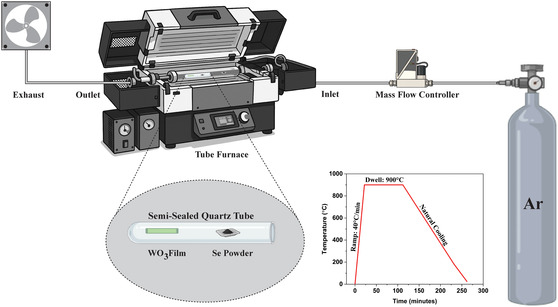
Schematic illustration of the hydrogen‐free APCVD‐assisted selenization of a predeposited WO_3_ thin film into WSe_2_, together with the corresponding temperature profile.

The edge‐enriched WSe_2_ films were synthesized through a two‐step process consisting of WO_3_ precursor film deposition, followed by hydrogen‐free APCVD‐assisted selenization. In the first phase, a highly doped Si substrate covered with a 285 nm SiO_2_ insulation layer was ultrasonically cleaned with acetone, ethanol, and deionized (DI) water for ten minutes consecutively. Then, WO_3_ films were directly grown onto SiO_2_/Si substrate by utilizing AACVD as depicted in Figure S1, based on our previous work [39]. In the second step, as‐grown WO_3_ films were selenized using our custom‐made tube‐in‐tube, hydrogen‐free, simple APCVD setup shown in Figure 1 [40]. The reaction took place in a dual‐zone quartz tube furnace, with a high‐temperature downstream zone (900°C) and a low‐temperature upstream zone (400°C). The temperature gradient of each zone was verified by thermocouples. Commercial Selenium (Se) powder (Thermo Scientific, CAS: 7782‐49−2) was used as a precursor. The substrate and one ceramic boat containing 350 mg Se powder were placed in a secondary semi‐sealed quartz tube, as depicted in Figure 1. This small tube was then positioned inside the primary quartz tube in the high‐temperature zone of the furnace. Another ceramic boat carrying the same amount of Se powder was kept outside the upstream zone in the primary quartz tube. Prior to selenization, the quartz tube was purged with 100 mL/min Argon gas for 1 h to eliminate the existing oxygen impurities. For selenization, the deposition temperature was set to 900°C with a ramp of 40°C/min. This optimized thermal exposure was empirically determined as the strict thermodynamic threshold required for the complete solid‐gas conversion of the WO_3_ precursor. Comprehensive gradient experiment tracking the structural evolution has been shown in Figure S2. When the temperature reached 900°C, the Se boat placed outside the upstream zone was moved in such a way that it changed its position to the 400°C upstream zone. The semi‐sealed secondary quartz tube moved over a few centimeters but remained in the 900°C downstream zone. The dwell time was set to 1.5 h to ensure the complete selenization of WO_3_ onto the substrate. The Ar flow of 30 mL/min was maintained during the whole deposition process. After the dwell time, the furnace was left to cool down naturally to the room temperature.

In conventional selenization of WO_3_, H_2_ serves as a chemical reducing agent that converts WO_3_ into oxygen‐deficient intermediates (WO_3‐x_) before Se can react with the tungsten center. Huang et al. explicitly demonstrated that no WSe_2_ is obtained when H_2_ is excluded from WO_3_ selenization under standard CVD conditions, establishing H_2_ as a thermodynamic necessity in the conventional route [41]. However, the key thermodynamic innovation in our approach lies in the engineering of a locally supersaturated Se atmosphere through the custom tube‐in‐tube semi‐sealed reactor design combined with a dual Se source configuration, which collectively fulfills the reducing role of H_2_ through a different thermodynamically equivalent pathway. The thermodynamic activity of Se vapor is sufficient to displace lattice oxygen from WO_3_ as volatile selenium oxides at 900°C, thereby progressively reducing from WO_3_ to WO_3‐x_ without requiring hydrogen. The formation of volatile SeO_2_ acts as the thermodynamic oxygen sink analogs to the role of H_2_O in the conventional H_2_‐assisted route, driving the equilibrium strongly toward WSe_2_ by continuously removing oxygen from the reaction zone. This mechanism is supported by the work of Exstrom and the coworkers, who showed that nonreducing selenization of WO_3_ is achievable when Se vapor has sufficient access to W atoms, as oxygen‐deficient WO_*x*_ sites lead to faster reactivity at elevated temperatures [42]. In our case, the semi‐sealed inner quartz tube traps Se vapor in the immediate vicinity of the WO_3_, ensuring a localized partial pressure far greater than that produced by conventional open‐tube configurations. This elevated Se chemical potential shifts the Gibbs free energy of the ${\mathrm{WO}}_{3}+\mathrm{Se}\;\to \;{\mathrm{WSe}}_{2}+{\mathrm{SeO}}_{2}$ reaction sufficiently negative to proceed without H_2_, satisfying the thermodynamic criterion ΔG < 0 at the surface [43]. Moreover, the placement of 350 mg Se in the semi‐sealed inner tube (at 900°C) and an additional 350 mg Se in the outer tube (repositioned to the 400°C upstream zone at the start of the dwell) ensures a continuous and replenished Se flux throughout the 1.5 h dwell. This extended dwell time at 900°C ensures sufficient kinetic energy to overcome the activation barrier of W—O bond breaking and W—Se bond formation without a chemical reducing agent [44]. The 1 h pre‐purging of Ar before deposition further removes residual O_2_, ensuring Se vapor does not compete with oxygen during selenization.

### 2.2 Structural Characterization

The surface morphology was examined using a field emission scanning electron microscope (FESEM, Thermo Scientific Scios 2 DualBeam) operating under high vacuum with an accelerating voltage of 5 kV. Cross‐sectional thickness analysis of the as‐deposited sensing film was performed via focused ion beam (FIB) milling. Prior to analysis, the targeted area was coated with a Pt layer to prevent surface damage during the milling process. The automated cross‐sectioning process was facilitated by AutoTEM software (Thermo Fisher Scientific) to ensure high precision and consistency across samples. Elemental composition of the active layer was determined by EDX coupled to the FESEM, and quantitative microanalysis was carried out using Pathfinder software. TEM and HRTEM analyses were performed with a JEOL F200 ColdFEG microscope operated at 200 kV. For TEM observation, the WSe_2_ film was mechanically scratched from the substrate, dispersed in absolute ethanol via sonication, and drop‐casted onto carbon‐coated copper grids. Raman spectroscopy was carried out using a Renishaw InVia spectrometer equipped with a 50× long working distance objective (NA = 0.75) and a 514 nm excitation laser. Crystal structure was investigated by XRD using a Bruker AXS D8 Advance diffractometer with a vertical theta–theta goniometer, incident and diffracted beam Soller slits of 2.5°, a fixed receiving slit of 0.5°, and an automatic air‐scattering knife. Cu Kα radiation was generated from a copper tube operating at 40 kV and 40 mA, and diffracted beams were detected using a LynxEye XE‐T PSD detector with an opening angle of 2.94°. Samples were mounted on a low‐background Si (510) holder. Diffractograms were analyzed using DIFFRAC.EVA 6.0 software together with the PDF‐2 (2022 release) database from the ICDD. XPS measurements were performed at SRCiT (URV) using a ProvenX‐NAP spectrometer equipped with a dual monochromatic X‐ray source (Al Kα = 1487 eV and Ag Lα = 2984 eV). Spectra were acquired at an X‐ray power of 100 W (Al Kα anode) with the hemispherical analyzer operating in fixed transmission mode, using a 7 × 20 mm entrance slit and an open exit slit with mesh. The beam spot size was approximately 200 µm. Data were collected with a PHOIBOS 150 NAP 1D‐DLD detector under a base pressure better than 2 × 10^−9^ mbar. Survey spectra were recorded at a pass energy of 80 eV and a step size of 0.7 eV, while high‐resolution spectra were acquired at 30 eV pass energy with a 0.3 eV step size to analyze chemical states and oxidation environments. Measurements were performed at a 0° emission angle relative to the surface normal, corresponding to an analysis depth of approximately 5–10 nm. Data processing was carried out using CasaXPS software (version 2.3.26), and elemental concentrations were calculated from peak areas using manufacturer‐provided sensitivity factors.

### 2.3 Sensor Fabrication and Measurement System

For our prototype sensor fabrication, the as‐grown WSe_2_ over SiO_2_/Si substrate was attached to an alumina holder where two Pt electrodes are screen printed. These electrodes are then directly connected to the substrate by platinum wires in order to complete the gas sensor circuit. The optical photograph of the fabricated sensor is shown in Figure S3. A customized gas detection system shown in Figure S4 was used for all the gas sensing measurements. The sensor was placed in a homemade Teflon chamber with an inner volume of 35 cm^3^, capable of accommodating up to four sensors simultaneously. A computer‐controlled mass flow system (EL‐Flow, Bronkhorst) provided a steady flow of gases into the inlet of the chamber at a rate of 100 mL/min, while the outlet was connected to the exhaust. Target NO_2_ concentrations were generated by dynamic dilution of the calibrated 1 ppm cylinder with dry synthetic air using the mass flow system. For a target concentration of C ppb, the NO_2_ flow rate was set to $Q_{{\mathrm{NO}}_{2}}=\frac{C}{1000}\times 100\mathrm{ml}/\min$ and the diluting synthetic air flow to $Q_{\mathrm{air}}=100-Q_{{\mathrm{NO}}_{2}}\mathrm{ml}/\min$, maintaining a constant total flow rate of 100 ml/min into the chamber all the times. Prior to each measurement sequence, the chamber was purged with at least three full volume exchanges to ensure complete removal of residual analyte gas. In addition, a controller evaporator mixer utilizing DI water (W‐202A, Bronkhorst) installed at the chamber's inlet to quantify the gas‐sensing performance in humid environment. A temperature and humidity sensors (SHT85, SENSIRION) were placed at the chamber outlet to monitor the real‐time environmental conditions. An Arduino‐based board was used to bridge the SHT sensor and the computer. For humidity‐dependent measurements, sensors were equilibrated at each RH level for a minimum of 2 h prior to data acquisition. The sensor resistance variations were monitored using an Agilent‐34972A Data Acquisition System and the data were stored by a software named Bench Link Datalogger 3 integrated to the data acquisition system. All gas sensing data are reported as the mean ± standard deviation from *n* = 3 independent sensor devices fabricated under identical conditions. For our experiments, the sensors were always tested at room temperature. The sensors were periodically exposed to the NO_2_ gas for 10 min, followed by 1 h under dry air to recover the baseline. The sensor responses were calculated using the Equation (1).

(1) $$\text{\%}\text{Response}=\frac{\left|R_{\mathrm{Air}}-R_{\mathrm{Gas}\;}\right|}{R_{\mathrm{Air}}}\times 100$$

where *R*_Gas_ and *R*_Air_ denote the resistance recorded in the target analyte and dry air, respectively.

### 2.4 Computational Methods

#### 2.4.1 First‐Principles Calculations

We employed DFT to simulate the adsorption of NO_2_ gas on 2H and 1T′ WSe_2_ substrates and compare their adsorbate configurations, energetics, and electronic structures. We employed the all‐electron, numeric atom‐centered orbital code FHI‐aims [44, 45] with the Perdew–Burke–Ernzerhof (PBE) exchange–correlation functional [45] nonlocal many‐body dispersion vdW corrections (MBDNL) [46, 47, 48, 49]. A tolerance for maximum residual force 5 × 10^−3^ eV/Å per atom and the charge density convergence criteria of 1 × 10^−6^ eV were used to relax monolayers of both the 2H and 1T′ phases of WSe_2_. For the optimization of the (1 × 1) unit cells, k‐space grids of 10 × 10 × 1 and 8 × 10 × 1 were employed for the 2H and 1T′ phases, respectively. Given convergence studies, we utilized “light” numerical settings for adsorption structure prediction and switched to “tight” settings for final structure relaxation and electronic structure calculations. Dense k‐space grids of 24 × 24 × 1 and 20 × 24 × 1 were used for band structure calculations of pristine 2H and 1T′ phases, respectively.

Supercell height of 30 Å was introduced in adsorption calculations to eliminate interactions between periodic images along the surface‐normal (z) direction. To model NO_2_ adsorption, a 4 × 4 × 1 supercell from the optimized 2H monolayer and a 3 × 4 × 1 supercell from the 1T′ phase were constructed to ensure negligible interactions between images of adsorbed molecules in in‐plane directions. For these adsorption calculations, we employed k‐space grids of 4 × 4 × 1 for the 2H and 2 × 4 × 1 for the 1T′ surfaces. The adsorption distance of the molecule and the surface was defined as the distance between the center of mass of NO_2_ and the surface formed by the topmost Se atom. The band edge energies used for the Schottky barrier analysis were obtained from fully spin‐polarized calculations.

**FIGURE 2:**
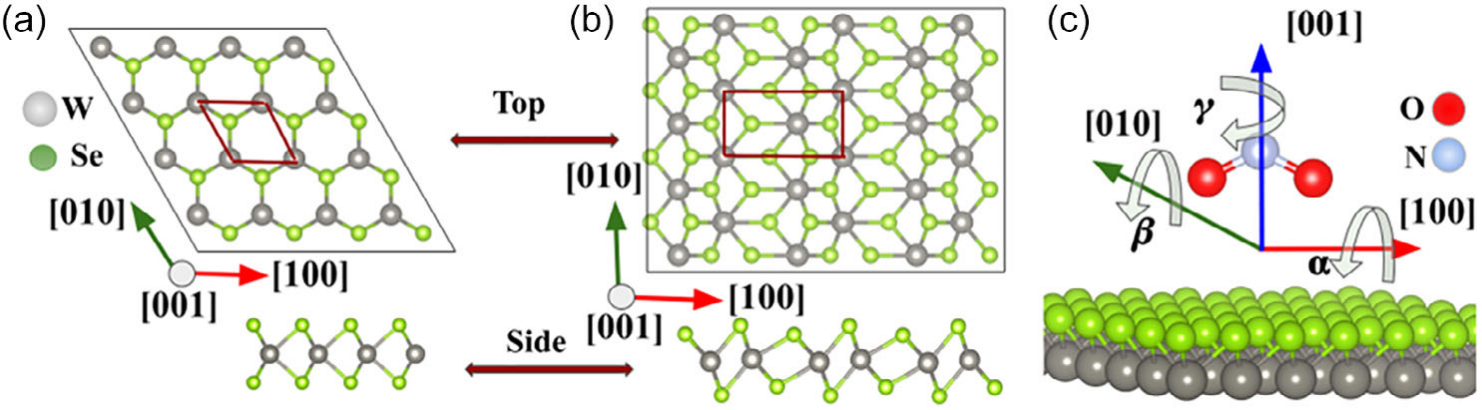
Supercell illustration of (a) 2H phase and (b) 1T′ phase of WSe_2_ monolayers in top and side views. The marked maroon area in both supercells was used for BOSS structure search. The 6‐dimensional degrees of freedom of the molecule used during the structure search are shown in (c).

The 2H phase of WSe_2_ crystallizes in the hexagonal space group *P6*_3_ */*mmc, which contains three atomic layers, with the W atoms located in the central layer and two symmetrically positioned Se atom layers above and below it, as shown in Figure 2a. The relaxed lattice constants of the unit cell are *a* = *b* = 3.29 Å. The optimized W—Se bond length is 2.54 Å and the W—Se—W bond angle is 83.35°. These structural parameters are in good agreement with previously reported experimental and computational studies [50, 51]. The PBE bandgap is calculated to be 1.65 eV, which matches perfectly with the value reported in Ref. [51]. Alternatively, the 1T′ phase exhibits a distorted octahedral with the monoclinic space group of P21/m. As shown in Figure 2b, the inequivalent vertical positions of Se atoms make its surface corrugated by 0.5 Å. The calculated lattice constants are *a* = 5.90 Å and *b* = 3.27 Å. Unlike the 2H phase, the bond lengths in the 1T′ are different, with the longest W—Se bond length being 2.64 Å. The PBE + SOC band gap is found to be 11 meV, showing the semi‐metallic characteristic of the 1T′ phase. These theoretical results are in good agreement with the previous studies [52, 53].

The adsorption energy of NO_2_ on the WSe_2_ monolayer was calculated using Equation (2).

(2) $$E_{\mathrm{ads}}=E_{\mathrm{tot}}-\left(E_{{\mathrm{WSe}}_{2}}+E_{{\mathrm{NO}}_{2}}\right)$$

Here, *E*_tot_ is the total energy of the combined materials, $E_{{\mathrm{WSe}}_{2}}$is the total energy of the isolated WSe_2_ monolayer, and $E_{{\mathrm{NO}}_{2}}$ is the total energy of the isolated NO_2_ molecule placed in the same supercell as all periodic simulations.

#### 2.4.2 Adsorbate Structure Identification

The most energetically favorable correct adsorption configuration of NO_2_ on the WSe_2_ surface requires examining all possible adsorption sites. To accelerate this search, we employed an active learning approach, Bayesian optimization structure search (BOSS) [54], to identify the lowest energy configuration on the adsorption energy surface (AES). In past research, BOSS efficiently identified the dominant adsorbates in gas and biosensors [40, 55].

We systematically leveraged BOSS to explore the movement of the center of mass of the NO_2_ molecule in the 6D phase space of adsorbate position and orientation. Three translational degrees of freedom were considered, where NO_2_ was confined within the x‐y plane, as mentioned in the maroon region in Figure 2a,b. Along the z‐direction, the molecule was allowed to move from 2.5 to 4 Å above the surface. Here, the x, y, and z directions correspond to the crystallographic [100,010] and [001] directions of the surface unit cell, respectively. Three rotational degrees of freedom (α, β, γ) of the NO_2_ molecule are responsible for the rotation of the molecule around the x‐, y‐, and z‐crystallographic axes, respectively, with all rotations confined to the range of 0°–360°. For translational motion in x and y directions and for all rotational degrees of freedom, we applied the standard periodic kernel. For the motion along the z‐direction, the radial basis function kernel (RBF) was used. Adsorption energetics were sampled using the exploratory Lower Confidence Bound (eLCB) acquisition function, which balances exploitation and exploration. The 6D BOSS active learning procedure was initiated with 30 Sobol space‐filling points, and iterations were continued until convergence to the global minimum configuration was achieved. The global minimum was found after approximately 1900 iterations for the 2H phase and 1300 iterations for 1T′ of WSe_2_. The BOSS predicted global minimum structure was fully optimized using “tight” settings of FHI‐aims, then subjected to electronic structure characterization.

## 3 Results and Discussion

### 3.1 Morphological and Elemental Analysis

**FIGURE 3:**
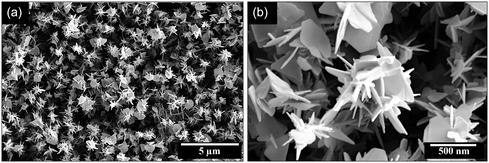
FESEM micrographs of (a) as‐grown WSe_2_ and (b) high magnification image showing the nano‐butterfly‐like shaped morphology.

The surface morphology of the as‐synthesized WSe_2_ film over the SiO_2_/Si substrate is illustrated in the FESEM micrographs shown in Figure 3. As shown in Figure 3a, the film exhibits a uniform, high‐density growth of WSe_2_ nanostructures across the substrate. A distinctive feature of this morphology is the presence of porous voids interspersed between the structural clusters. During the reaction at 900°C, the substitution of oxygen in the solid WO_3_ precursor by selenium generates volatile SeO_2_ byproducts. The rapid sublimation of SeO_2_ created this observed void, which serves a critical functional role by providing diffusion channels for target gas molecules to penetrate deeper into the sensing interface [56]. The high magnification FESEM micrograph in Figure 3b reveals the detailed architecture of the film, characterized by vertically aligned “nano‐butterfly” shaped triangular assemblies. These structures have an average lateral dimension of approximately 400 nm. The vertical orientation arises from the crowding effect inherent to the conversion of a thick precursor film [57]. The high nucleation density restricts lateral expansion on the basal plane, forcing the WSe_2_ sheets to grow out‐of‐plane to access the supersaturated Se vapor flux supplied by the semi‐sealed tube. The unique butterfly‐like geometry is attributed to crystallographic twinning during the initial nucleation stage [58]. At the elevated synthesis temperature of 900°C, adjacent triangular nuclei merged along rotational twin boundaries. Driven by anisotropic kinetics, the chemically active edges of these twin crystals grew rapidly outward, forming the sharp, wing‐like facets observed in the micrograph. Furthermore, a comprehensive spatially resolved FIB‐assisted cross‐sectional thickness analysis was carried out on three independently fabricated sensor films. As depicted in Figure S5, the thickness values were consistently found within the range of approximately 3.9 to 4.4 µm, demonstrating good spatial uniformity of the active sensing layer across different devices. Such thickness uniformity is important for maintaining reproducible gas diffusion pathways and charge‐transport characteristics, which is consistent with the good device‐to‐device reproducibility observed later in the sensing response [59]. The elemental studies by EDX, shown in Figure S6, reveal the strong presence of W and Se. Minimal carbon impurity was observed, which was attributed to the residual organic precursors used during the synthesis process. The quantitative microanalysis of the elements is presented in Table S1 in detail. The atomic ratio between the W and Se was found to be 1:2, which is consistent with the expected stoichiometry [60].

### 3.2 Structural and Crystallographic Studies

**FIGURE 4:**
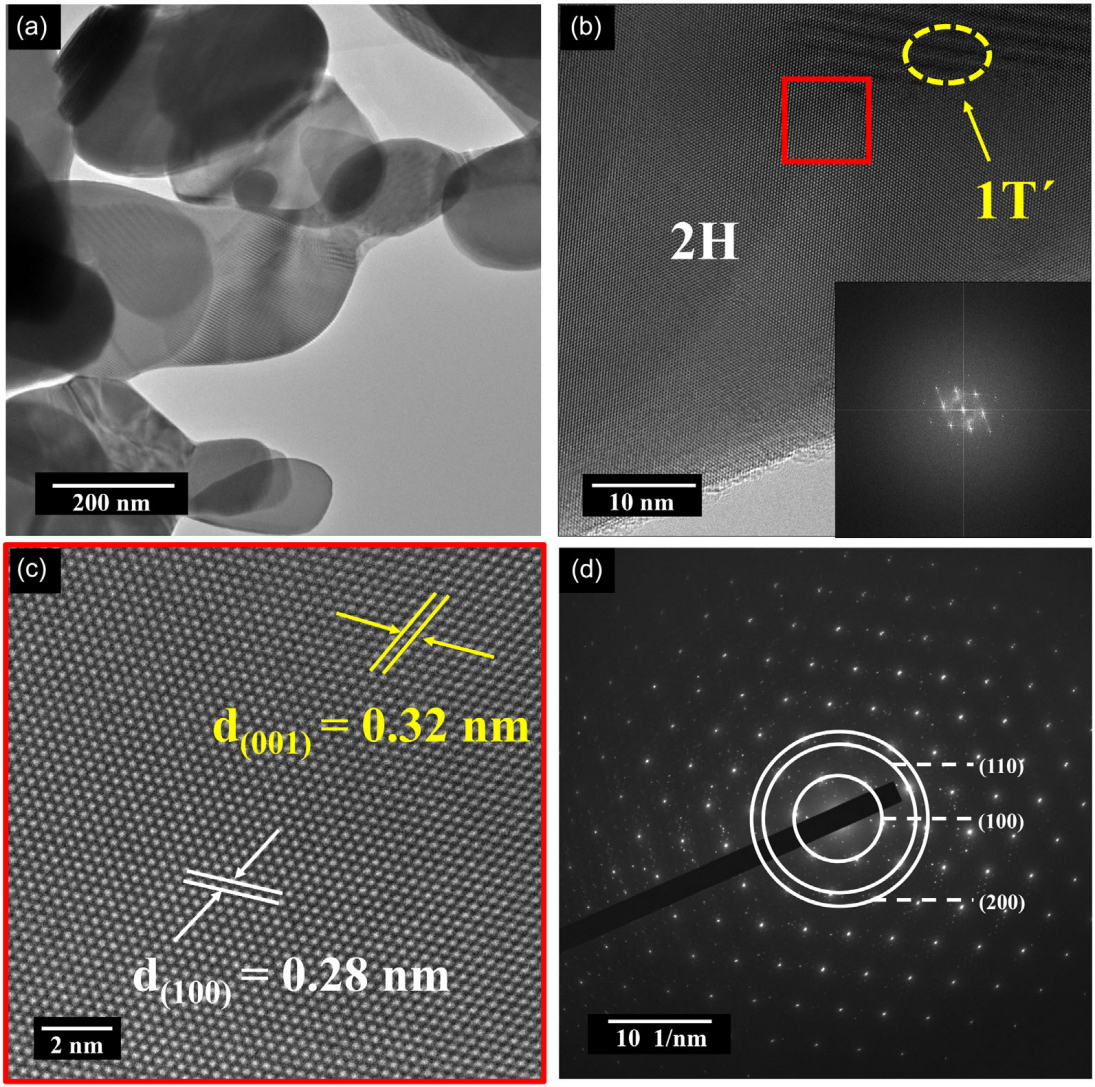
(a) Low magnification TEM image of WSe_2_ triangular petals, (b) HRTEM image showing coexistence of dual phase, corresponding FFT in inset image, (c) interplanar distances along crystal planes at atomic scale in the red marked area in (b) and (d) corresponding SAED pattern showing highly ordered diffraction spots.

The morphology was further examined by low magnification TEM analysis, as illustrated in Figure 4a. The presence of large, thin, and transparent triangular petals is consistent with the results obtained from FESEM. These triangular sheets are arranged on top of one another and form nano‐butterfly‐like shape as observed by FESEM. Structural analysis at the atomic scale was further explored in depth by HRTEM. Figure 4b shows multiple crystalline layers with visible parallel lattice fringes. This prevailing honeycomb‐like pattern represents the semiconducting 2H phase of WSe_2_ [41]. Another small region of contrasting zigzag chains of tungsten atoms (marked in yellow) is ascribed to the metastable 1T′ phase of WSe_2_ [61, 62]. In the inset image, the FFT pattern clearly exhibits the hexagonal symmetry with six bright spots arranged around the center. The interplanar distance along the (100) crystallographic plane of 2H‐WSe_2_ was found to be 0.28 nm, as depicted in Figure 4c, which is consistent with the existing literature [24, 63]. On the other hand, the lattice spacing for 1T′‐WSe_2_ along the (001) plane was calculated as 0.32 nm [64]. The SAED pattern in Figure 4d exhibits hexagonally arranged diffraction spots, confirming its high crystallinity. By evaluating the reciprocal interplanar d‐spacings along the [001] zone axis, the prominent rings were indexed to the (100), (110), and (200) crystallographic planes of the dominant 2H phase. Apparently, a coexistence of a dominant semiconducting phase along with a secondary semimetallic phase was found, which is further validated by the XRD, Raman spectroscopy, and XPS [65]. These heterophase boundaries create additional defects acting as preferential adsorption centers for target gas molecules and consequently amplify the sensor response [66].

**FIGURE 5:**
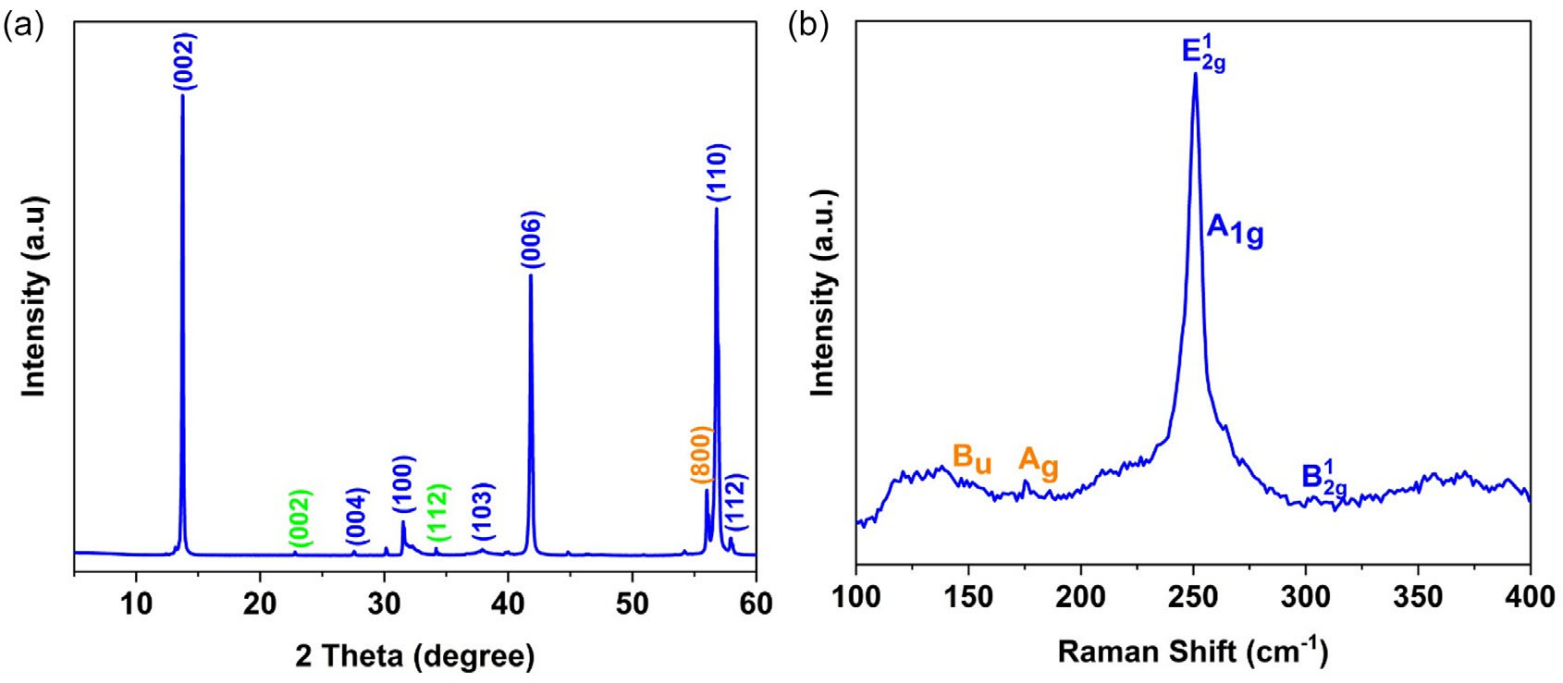
(a) XRD diffractogram of as‐grown WSe_2_ and (b) Raman spectral analysis of WSe_2_.

The crystallinity and phase purity of the WSe_2_ film were further investigated by XRD. Figure 5a displays the XRD diffractogram of as‐synthesized WSe_2_ over SiO_2_/Si substrate. The characteristic diffraction peaks at 13.6°, 27.5°, 31.4°, 37.8°, 41.7°, 47.3°, 55.9°, and 57.9° correspond to (002), (004), (100), (103), (006), (105), (110), (112) crystal planes (marked in blue) of the 2H phase WSe_2_, respectively (ICDD card no. 01‐071−0600). The sharp, intense peak with narrow full width at half maximum (FWHM) indicates a highly ordered crystalline phase, implying crystallites larger than 400 nm [67]. Another less prominent reflection from the 1T′ phase at 56.1° was identified along the (800) crystal plane (marked in orange) [68]. In addition, marginally present diffraction peaks of WO_3_ were observed at 22.8° and 34.2° ascribed to (002) and (112) crystal planes (marked in green) respectively (ICDD card no. 00‐043−1035).

### 3.3 Raman Spectroscopic Analysis

The vibrational properties and structural fingerprint obtained by Raman spectroscopy in the 100–400 cm^−1^ region are exhibited in Figure 5b. Two distinct characteristic Raman peaks of semiconducting 2H‐WSe_2_ have been observed. The prominent peak at 251 cm^−1^ corresponds to the $E_{2g}^{1}$ in‐plane vibration of W and Se atoms, whereas a shoulder peak appearing at 258 cm^−1^ ascribes to $A_{1g}$ out‐of‐plane vibration of Se atoms [69, 70]. The frequency difference between these two characteristic phonon modes was found to be 7 cm^−1^, and the FWHM of $E_{2g}^{1}$ was determined around 2 cm^−1^, indicating multilayer WSe_2_ [71]. Another low intensity peak at 304 cm^−1^ corresponds to $B_{2g}^{1}$ probing the interlayer interaction and symmetry breaking in few‐layer WSe_2_ [41]. Additionally, two less prominent peaks at 149 and 177 cm^−1^ are ascribed to the B_u_ and A_g_ modes, respectively, associated with the 1T′ phase. Similar results have been reported in some of the existing reports [61, 62, 68, 72].

### 3.4 Chemical State Analysis

**FIGURE 6:**
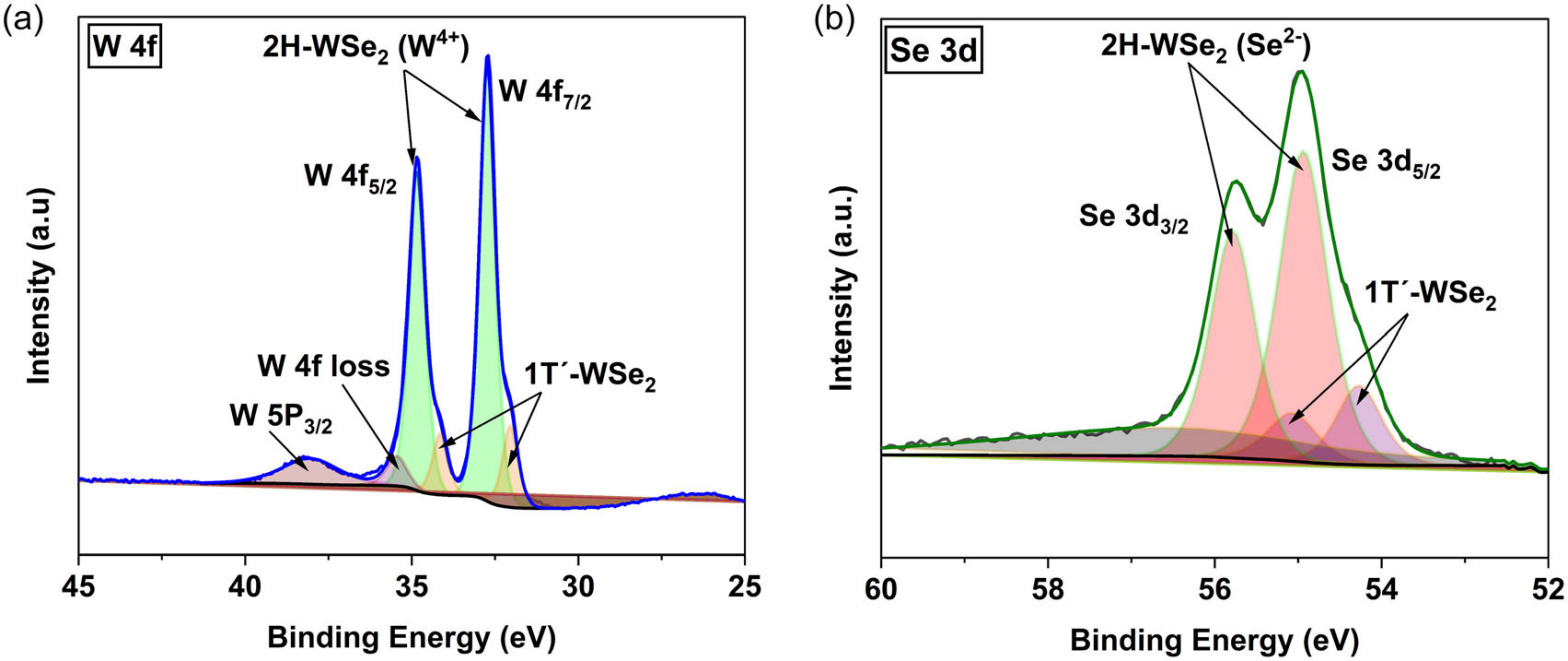
XPS spectra of as‐synthesized WSe_2_. (a) W 4f core level and (b) Se 3d core level.

XPS‐based elemental and chemical state investigation of as‐grown WSe_2_ is displayed in Figure 6. The elemental survey scan confirms the presence of W, Se, O, and C as shown in Figure S7. The core‐level XPS spectrum has been recorded for W 4f and Se 3d binding energy regions. The W 4f spectrum is composed of 2 main peaks, which are deconvoluted into two doublets and one small peak. As depicted in Figure 6a, the dominant 2H‐WSe_2_ phase (green shaded peaks) confirms the successful synthesis of semiconducting WSe_2_ via APCVD. The predominant doublet features at 32.7 and 34.8 eV are assigned to the W^4+^ oxidation state, corresponding to the W 4f_7/2_ and W 4f_5/2_ spin–orbit components, respectively [73, 74, 75]. For accurate deconvolution of the W 4f spectrum, the intrinsic W 5p_3/2_ feature, consistent with the W^4+^ chemical state, was included in the fit at 38.2 eV. The second doublet located at 31.9 and 34.0 eV indicates a minor contribution from the 1T′ WSe_2_ phase, as reported in [61]. This partial phase transformation at interfaces or edges exhibits a characteristic 0.8 eV shift to the lower binding energy compared to the 2H phase. Due to the W 4f loss, an additional peak at 35.5 eV has appeared [67]. On the other hand, the Se 3d spectra show characteristic doublet splitting with the dominant component from 2H‐WSe_2_ (red shaded peaks) as shown in Figure 6b. The presence of 1T′‐WSe_2_ selenium signals at slightly lower binding energies confirms the coexistence of both phases, consistent with the W 4f analysis. The 1T′Se^2−^ doublet appears at 54.3 and 55.1 eV, while the 2H Se^2^^−^ doublet is at higher binding energies (54.8 and 55.6 eV). Moreover, the W/Se stoichiometry ratio was found to be 1:2, which is consistent with the EDX results. Quantitative peak area analysis of the deconvoluted W 4f and Se 3d core levels reveals that the metallic 1T′ phase constitutes approximately 15.5% and 18.9% of the surface composition, respectively, with the semiconducting 2H phase comprising the dominant remaining fraction (∼81–84%). The XPS‐derived phase fractions are summarized in Table S2.

### 3.5 Gas Sensing Results at Ambient Temperature

**FIGURE 7:**
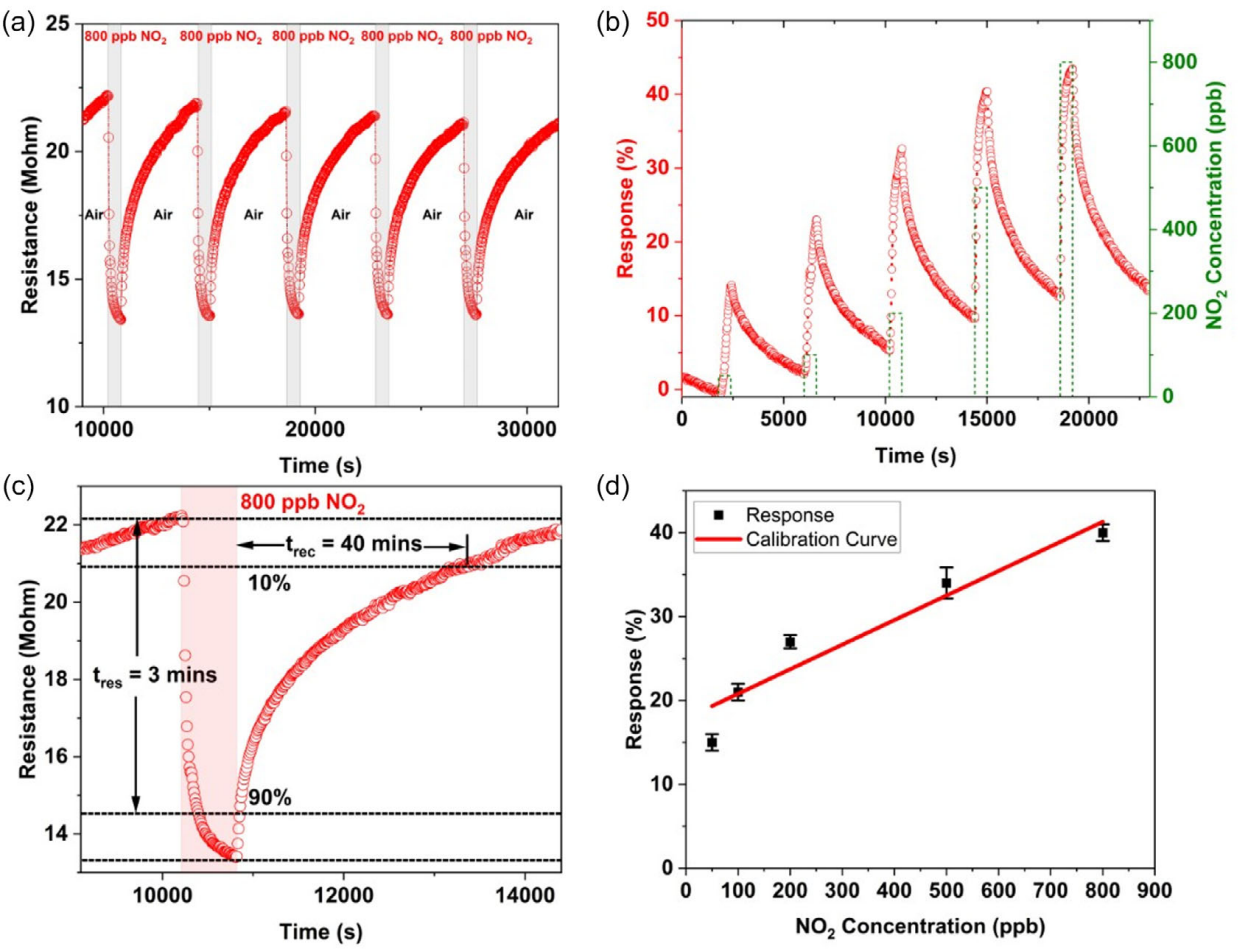
Gas sensing in dry conditions. (a) Sensor resistance dynamics for 800 ppb NO_2_, (b) change of sensor responses as a function of time toward different NO_2_ concentrations at room temperature, (c) quantification of response and recovery times for 800 ppb NO_2_, (d) calibration curve obtained from different NO_2_ concentrations. Error bars represent the mean ± standard deviation derived from *n* = 3 independent sensors.

The gas‐sensing characteristics of our WSe_2_ sensor were systematically evaluated by tracking its chemiresistive response upon exposure to varying concentrations of NO_2_ at 25°C. At the outset, the sensor was subjected to multiple cycles of 800 ppb NO_2_ as depicted in Figure 7a. Upon exposure to the oxidizing gas NO_2_, a decrease in resistance confirms its p‐type semiconducting nature, with holes as the majority charge carriers. The sensor exhibited a remarkable response of 40% along with a full recovery to the baseline. Furthermore, minimal variation (around 1%) in response magnitude across multiple exposure cycles was observed, validating the sensor's stable detection performance. Hence, the sensor demonstrated high repeatability, which is a crucial parameter for reliable gas sensing [76].

Figure 7b illustrates the evolution of the sensor response upon exposure to sequentially increasing NO_2_ concentrations ranging from 50 ppb to 800 ppb. The sensor was operated at room temperature. The sensor responses follow a linear trend with increasing NO_2_ concentrations at room temperature. Evidently, this distinct concentration‐dependent response highlights the outstanding sensitivity of our sensor over a broad dynamic range. In addition, repeatability tests for all concentration levels were performed individually. The corresponding results are presented in Figure S8, endorsing excellent reproducibility.

Response time and recovery time are two key metrics for assessing gas sensing performance under ambient conditions. The response time (*t*_res_) is defined as the time taken to reach 90% of the maximum response during target gas exposure, while the recovery time (*t*_rec_) corresponds to the time required to return to 10% of the maximum response upon subsequent exposure to dry air. Figure 7c shows the quantification of the response and recovery times subjected to 800 ppb NO_2_. The sensor took 3 min to reach 90% of the maximum response while the recovery time was 40 min, demonstrating commendable performance relative to previously reported WSe_2_‐based sensors operating at room temperature [30, 77]. The prolonged response and recovery times at room temperature are primarily attributed to the high adsorption energy of NO_2_ molecules over the WSe_2_ surface, impeding the rapid adsorption and desorption processes [78]. Besides, they are highly related to the flow rate used during the gas sensing measurements. Indeed, a low gas flow rate results in slow detection kinetics. In this work, we used 100 mL/min as mentioned in the experimental part. Applying external stimuli (e.g., photoactivation or elevated operating temperature), surface functionalization, and heterojunction formation could be some potential mitigation strategies in order to accelerate both adsorption and desorption kinetics [21, 79, 80, 81].

Figure 7d demonstrates the calibration curve rendered from the sensor responses toward NO_2_ concentrations ranging from 50 to 800 ppb, using a linear regression model. Regression analysis from the calibrated data yielded a Pearson correlation coefficient (*R*) of 0.95 and a squared correlation coefficient (*R* ^2^) of 0.91, reflecting high accuracy as well as reliability in the gas sensing measurements [82]. The sensitivity (S) was found as 0.029% ppb^−1^ from the slope of the calibration curve. In accordance with the International Union of Pure and Applied Chemistry (IUPAC) standards, a rigorous methodological framework was applied to calculate the theoretical limit of detection (LOD) [83]. The theoretical LOD is given by the Equation (3) is as follows

(3) $$\mathrm{LOD}\;=\;\frac{k\times s_{B}}{S}$$

where $s_{B}$ is the standard deviation of the sensor baseline percentage response determined from 200 baseline data points acquired prior to NO_2_ exposure ($s_{B}$ = 0.035%), *k* = 3 is the IUPAC recommended numerical factor, and *S* = 0.029% is the analytical sensitivity defined as the slope of the linear calibration curve.

**FIGURE 8:**
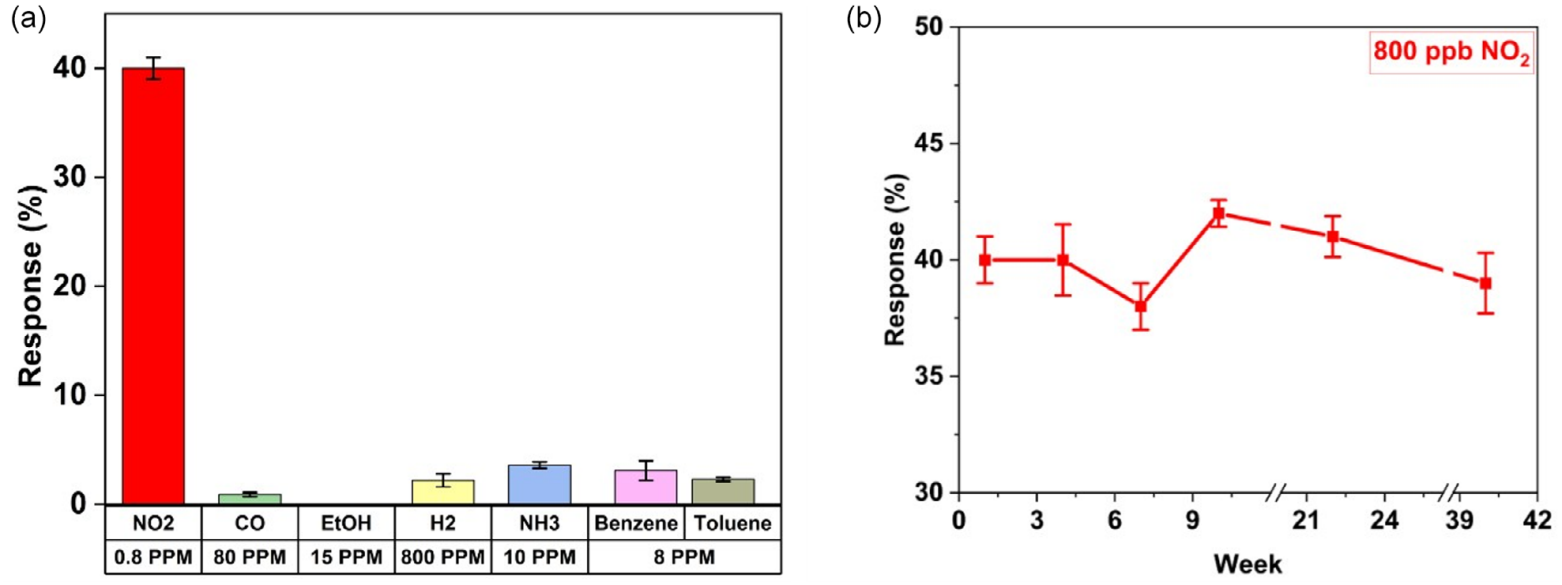
(a) Cross‐sensitivity test results and (b) sensor stability for 40 weeks. Error bars represent the mean ± standard deviation derived from *n* = 3 independent sensors.

The selectivity test for our sensors has been performed by exposing them to various reducing gases. In this regard, 80 ppm of carbon monoxide (CO), 800 ppm of hydrogen (H_2_), 10 ppm of ammonia (NH_3_), and volatile organic compounds (VOCs) such as 15 ppm of ethanol (C_2_H_6_O), 8 ppm of benzene (C_6_H_6_), 8 ppm of toluene (C_7_H_8_) have been tested based on their permissible toxicity level in the atmosphere. As depicted in Figure 8a, the sensor exhibited negligible responses to the aforementioned interfering gases in comparison to 800 ppb of NO_2_, validating its minimal cross‐sensitivity. Clearly, the selectivity test confirms superior specificity for trace‐level detection of NO_2_, even in the presence of far higher concentrations of common environmental pollutants. Although mixed‐gas selectivity testing would provide a more realistic assessment of cross‐sensitivity, such testing was beyond the capability of our present gas‐delivery system. Nevertheless, single‐interferent exposure remains a standard first‐level approach for evaluating cross‐sensitivity in chemiresistive gas sensors [84, 85]. Therefore, this robust selectivity ensures reliable NO_2_ monitoring in both indoor and outdoor environments.

The long‐term stability of the WSe_2_ sensors was further evaluated by periodically measuring their response to 800 ppb NO_2_ over 40 weeks. As shown in Figure 8b, the sensor response remained highly stable within the range of approximately 38–41% throughout the entire storage period, confirming excellent retention of sensing performance during long‐term aging. To assess operational robustness, comparative resistance transients recorded at week 1, week 10, week 20, and week 40 are presented in Figure S9. Although a slight increase in baseline resistance was observed with storage time, the transient profile, response magnitude and recovery behavior remained largely preserved. Furthermore, the corresponding response and recovery behavior depicted in Figure S10 showed only minor variation over the full 40 week period, indicating stable sensing kinetics. Moreover, device‐to‐device reproducibility tests (Figure S11) validated the reliability of our fabrication process for actual real‐world deployment.

### 3.6 Gas Sensing in a Humid Environment

**FIGURE 9:**
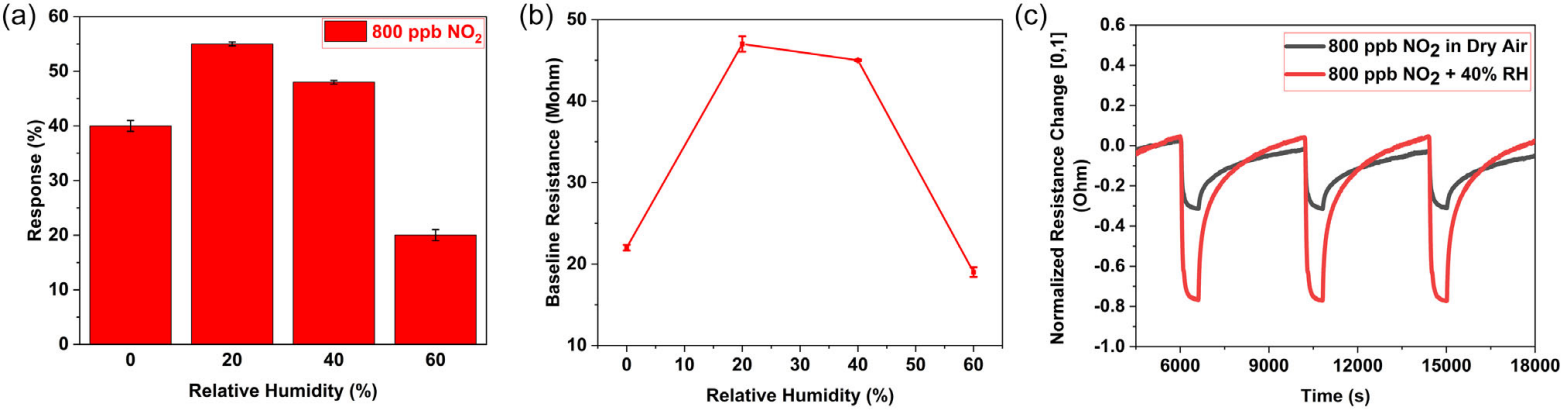
Gas sensing in a humid environment (a) response dynamics for different RH levels, (b) evolution of baseline resistance as a function of RH, (c) comparison of the responses toward 800 ppb NO_2_ for sensors operated at room temperature under dry and humid conditions; the sensor resistance changes are normalized to [0,1]. Error bars represent the mean ± standard deviation derived from *n* = 3 independent sensors.

Gas sensors operating at room temperature are particularly susceptible to interference from ambient humidity, primarily due to the insufficient desorption of water molecules in the absence of thermal assistance [86]. The facile adsorption of moisture on the sensing surface significantly hampers both sensitivity and recoverability [87]. Therefore, the gas sensing performance as well as the consequent variation in baseline resistance were systematically evaluated under various humid conditions and compared with the benchmarked results obtained in dry conditions. Our sensors were tested toward 800 ppb NO_2_ for several relative humidity (RH) levels ranging from 20% to 60% RH, as depicted in Figure 9a. An increment in the sensor response was observed initially, followed by a gradual diminution with elevated humidity levels. The sensor responses were recorded as 55%, 48%, and 20% for the RH of 20%, 40%, and 60%, respectively. Repeatable cycles of 800 ppb NO_2_ for these humid conditions are shown in Figure S12.

The enhanced response at lower humidity (20% RH) can be attributed to the synergistic effects of water vapor on gas adsorption and charge transfer mechanisms. At 20% RH, water molecules partially chemisorb and dissociate on the heterophase WSe_2_ surface, generating hydroxyl (OH^‐^) and proton (H^+^) species. The electron‐donating character of OH^−^ reduces the hole concentration in the *p*‐type WSe_2_ lattice, which is directly evidenced by the increase in baseline resistance at 20% RH (Figure 9b), consistent with previously reported behavior for *p*‐type two‐dimensional materials [88, 89]. However, it is critical to distinguish this bulk carrier modulation from the competing surface adsorption enhancement mechanism. The surface‐bound OH groups increase the local electron density at the WSe_2_ surface, creating electron‐rich secondary active sites that significantly facilitate the chemisorptive interaction with NO_2_ [90]. In this context, the OH‐mediated pathway amplifies the charge transfer from the sensor surface to NO_2_ molecules, generating a net enhancement in the *p*‐type response that exceeds the competing bulk hole reduction effect. As a result, a 37.5% increment in response is observed relative to dry conditions. At moderate humidity (40% RH), partial surface site saturation by water molecules progressively attenuates the OH‐mediated adsorption enhancement, yielding a diminished but still positive 20% augmentation in response (Figure 9c). At 60% RH, the formation of a continuous multilayer water film initiates protonic conduction through the Grotthuss mechanism (H_2_O + H_3_O^+^ → H_3_O^+^ + H_2_O), which shifts the dominant charge transport pathway from electronic to protonic [91]. The resulting proton hopping reduces the sensor resistance independently of NO_2_ exposure and concurrently blocks active adsorption sites, collectively producing a 20% reduction in sensor response relative to dry conditions (Figure 9b) [92, 93]. Furthermore, several anti‐humidity strategies such as surface engineering, physical isolation by waterproof membranes, working parameter modulation, algorism compensation, novel material development, etc., can be implemented to mitigate high humidity interference [94].

A rigorous comparison of the gas sensing performance between our heterophase WSe_2_ nano‐butterflies and recently reported state‐of‐the‐art WSe_2_‐based sensors is detailed in Table 1. Notably, most of these sensors were subjected to the higher concentrations of NO_2_, which is beyond the permissible limit of exposure as discussed in the earlier section. Also, some sensors only responded at elevated temperature, which can deteriorate the sensing material, affecting their sensitivity, stability, and selectivity. Even, some sensors were not tested under several RH conditions for evaluating their applicability in real‐world applications. Moreover, recent advancements in contemporary TMD‐based NO_2_ sensors have achieved high sensitivity through complex multicomponent hybridization, such as ternary composites or carbon‐nanotube networks (e.g., SnS_2_/MWCNT, WS_2_/WO_3_‐graphene), or require external UV activation to accelerate recovery kinetics [36, 37]. In contrast, our engineered 2H/1T′ WSe_2_ nano‐butterflies achieve an exceptional 40% response at a sub‐ppm level (800 ppb) entirely at room temperature. This demonstrates that highly competitive sub‐ppm detection and robust environmental stability can be achieved relying solely on intrinsic structural hierarchy and phase synergies, representing a meaningful advancement over conventional binary chalcogenide systems.

**TABLE 1:** Comparison of NO_2_ sensing performance for various WSe_2_‐based gas sensors.

Sensing film	Synthesis	Conc. (ppm)	T, °C	Response, %	**LOD,** **ppb**	Humidity test	Ref.
Trilayer WSe_2_	ALD	500	RT	4140	—	Not Available	[95]
WSe_2_	CVD	1	250	13.45	—	Not available	[26]
WSe_2_ nanosheets	LPE	10	RT	5.36	8	Heavily Affected	[23]
3D‐WSe_2_	CVD	1	RT	52.24	—	Moderately Affected	[65]
WSe_2_ nanosheets	LPE	0.05	RT	5.06	50	Heavily Affected	[24]
WSe_2_ nanoflowers	CVD	0.8	100	20.5	100	Moderately Affected	[30]
WSe_2_ concentric nanotriangle	CVD	20	RT	84	76	Heavily Affected	[27]
Porous 3D WSe_2_	CVD	10	150	33.3	800	Not available	[96]
Au decorated WSe_2_	LPE	5	RT	170	—	Moderately Affected	[97]
Pt decorated WSe_2_	LPE	5	RT	220	—	Moderately Affected	[97]
NbSe_2_/WSe_2_	CVD	5	RT	30	—	Moderately Affected	[98]
WSe_2_/MWCNT	Sonication	10	100	50	2.8	Moderately Affected	[35]
WSe_2_/WS_2_	CVD	10	RT	178	—	Not available	[99]
MoS_2_/WSe_2_	CVD	50	RT	15	—	Not available	[99]
WSe_2_ vertical nanosheets	CVD	1	RT	34.6	4	Moderately Affected	[63]
WSe_2_/SnS	LPE	0.025	RT	1.08	10.6	Heavily Affected	[100]
**WSe**_**2**_ **nano‐butterfly**	**CVD**	**0.8**	**RT**	**40**	**4**	**Moderately Affected**	**This Work**

Abbrevations: ALD: Atomic Layer Deposition, CVD: Chemical Vapor Deposition, LPE: Liquid Phase Exfoliation, RT: Room Temperature.

### 3.7 Computational Insights on NO_2_–WSe_2_ Interactions

#### 3.7.1 Adsorption of NO_2_ to 2H and 1T′ Surfaces of WSe_2_

**FIGURE 10:**
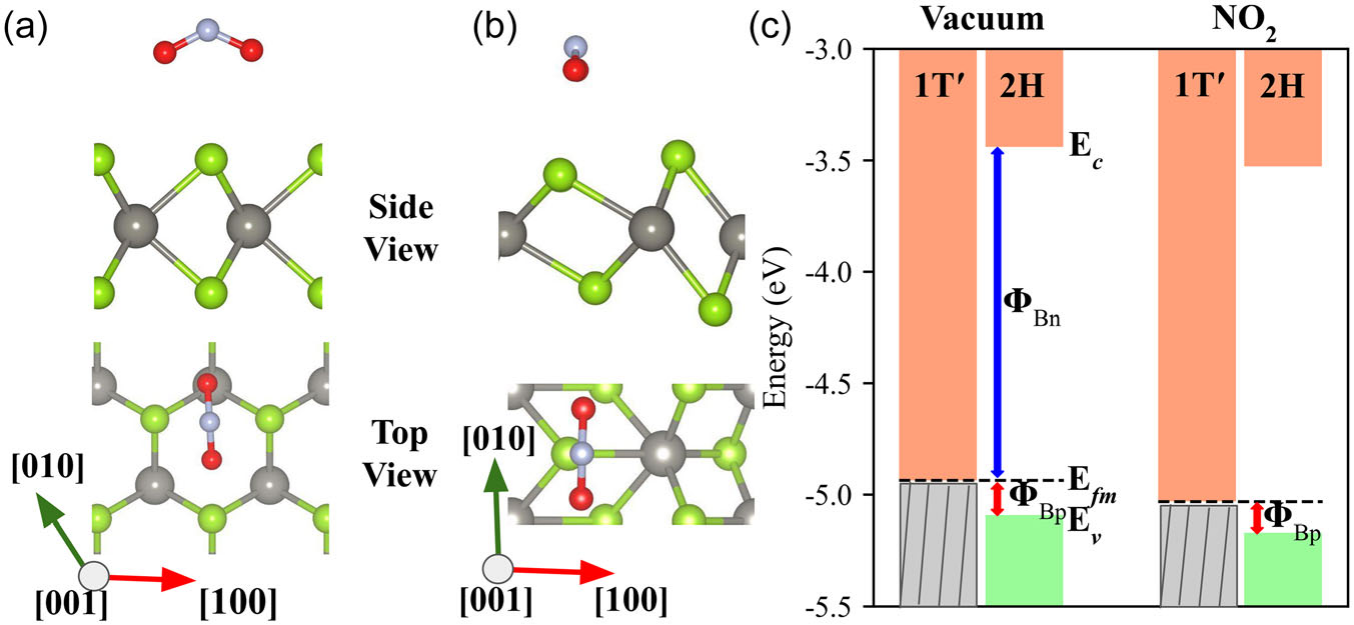
Adsorption configurations of NO_2_ are shown from the top and side views for (a) the 2H and (b) the 1T′ of WSe_2_. The energy band diagrams illustrating the band alignment of both the pristine and NO_2_‐adsorbed WSe_2_ phases are presented in (c). The left panel corresponds to the interface formed between pristine 1T′ and 2H monolayers, while the right panel shows the interface where NO_2_ is adsorbed on both surfaces. The Fermi energy (*E*_fm_) of semimetallic 1T′‐WSe_2_ is indicated by black dashed lines. The valence band maximum (*E*_v_) of 2H‐WSe_2_ is shown using light green bars, whereas the conduction band minimum (*E*_c_) is shown in red bars. All energy levels are referenced to the vacuum level set at 0 eV. The n‐type Schottky barrier (Φ_Bn_) and p‐type Schottky barrier (Φ_Bp_) are indicated with a blue arrow and a red arrow, respectively.

BOSS adsorbate structure search yielded a global minimum configuration of the NO_2_ adsorbed on 2H‐WSe_2_ with the center of mass of the NO_2_ at the position *x* = 7.10 Å, *y* = 0.66 Å, *z* = 3.43 Å, and with surface orientation angles *α* = −2.57°, *β* = −2.75°, *γ* = 58.61°. After the subsequent structural relaxation, we observed minimal changes in both the structural and energetic properties. The adsorption energy was calculated to be −0.31 eV. The adsorption site is the bridge position between two Se atoms, as shown in Figure 10a at a distance *d* = 2.9 Å from the surface.

For the 1T′ phase, as shown in Figure 10b, the preferred adsorption site is on top of the low‐lying Se atom with both oxygen atoms of NO_2_ oriented toward the surface. Downward orientation of O atoms is observed for both surface phases; however, molecular O atoms on the 1T′ phase are rotated toward Se atoms, in contrast to the clear W association on the 2H phase. The adsorption energy is −0.48 eV, which is lower than that of the 2H phase. In addition, the adsorption distance *d* = 2.3 Å is smaller than the adsorption distance on the 2H phase. A comparison of the structural properties of NO_2_ adsorption configurations on the two phases in Table 2 clearly indicates stronger binding on the 1T′ phase compared to the 2H phase.

**TABLE 2:** Adsorption energy *E*_ads_, adsorption distance d, and charge transfer *δq* of NO_2_ adsorption on two phases of WSe_2_ monolayer.

Surface	***E***_**ads**_	d, Å	*δq*, e
2H WSe_2_	−0.31	2.9	0.15
1T′ WSe_2_	−0.48	2.3	0.16

#### 3.7.2 Electronic Structure

To quantify the adsorption‐induced charge transfer between NO_2_ and the surface, we performed Mulliken charge analysis. For both structural phases, charge is transferred from the WSe_2_ surface to the NO_2_ molecule, confirming the acceptor nature of NO_2_ as an electron‐withdrawing adsorbate. The partial charge *δq* for both phases is tabulated in Table 2. For the semiconducting 2H phase, the charge transfer (*δ* *q* = 0.15 e) is close to the semi‐metallic 1T′ (*δ* *q* = 0.16 e). Both the adsorption energy and value of *δq* for 2H‐WSe_2_ match previous studies [101], but this is the first time adsorption on the 1T′ phase has been reported.

To understand the nature of the molecule–substrate bond, we explored the partial density of states (PDOS) for the adsorbate (not shown here). In both structural phases, the electronic states of NO_2_ remain as sharp as in the gas phase, which suggests that physisorption is the dominant adsorption mechanism. Minor hybridization of states in the 1T′ phase does point to weak chemical bond formation, consistent with the stronger adsorption of NO_2_ on this phase.

To examine Schottky barrier formation at the interface between the semi‐metallic 1T′ and semiconducting 2H phases of WSe_2_, both in air and in the presence of NO_2_, we calculated the electronic band energies of pristine and NO_2_‐adsorbed configurations. The resulting band positions − valence band maximum (*E*_v_), conduction band minimum (*E*_c_), and Fermi energy (*E*_f_), along with the band gap (*E*_g_), work function (*ϕ*), electron affinity (χ), and Schottky barrier heights (Φ) are summarized in Table 3. All the energies are referenced to the vacuum level at 0 eV. The corresponding band edge alignment at the 1T′–2H interface, in vacuum and in the presence of NO_2_, is illustrated in Figure 10c. The bandgap of both semiconducting 2H‐WSe_2_ and semi‐metallic 1T′‐WSe_2_ remains unchanged before and after NO_2_ adsorption. The calculated work function of semi‐metallic 1T′‐WSe_2_ (4.95 eV) differs from the electron affinity of semiconducting 2H–WSe_2_ (3.44 eV), leading to the formation of a Schottky barrier at the 1T′–2H interface. The energy difference between the conduction band minimum (*E*_c_) of 2H phase and the Fermi level of semi‐metallic 1T′ (*E*_fm_) defines the n‐type Schottky barrier (Φ_Bn_ = *E*_c_[2H] − *E*_fm_), whereas the p‐type Schottky barrier (Φ_Bp_ = *E*_fm_ − *E*_v_[2H]) is computed from the energy difference between E_fm_ of 1T′‐WSe_2_ and the valence band maximum of 2H‐WSe_2_. The band alignment computed here results in a p‐type Schottky barrier (Φ_Bp_) of 0.15 eV and a comparatively larger electron barrier (Φ_Bn_ 1.5 eV). A smaller Φ_Bp_ compared to Φ_Bn_ implies that the clean 1T′‐WSe_2_ and 2H‐WSe_2_ surfaces form a p‐type Schottky contact at the interface. The NO_2_‐adsorbed 1T′‐2H interface continues to exhibit a p‐type Schottky contact with a minimally reduced barrier (0.14 eV) compared to the pristine interface. Given the exponential dependence of Schottky transport on the barrier height, even a small reduction of 0.01 eV in the barrier height might lead to an increase in conductivity.

**TABLE 3:** Calculated electronic energy parameters (in eV) for pristine and NO_2_ adsorbed 1T′ and 2H WSe_2_, including valence band maximum (*E*_v_), Fermi energy (*E*_f_), conduction band minimum (*E*_c_), band gap (*E*_g_), work function (*ϕ*), electron affinity (χ), electron Schottky barrier height (Φ_Bn_) and hole Schottky barrier height (Φ_Bp_).

Configurations	*E*_v_	*E*_f_	*E*_c_	*E*_g_	*ϕ*	*χ*	Φ_Bp_	Φ_Bn_
1T′‐WSe_2_	−4.94	−4.94	−4.94	0.00	4.94	—	0.15	1.5
2H‐WSe_2_	−5.09	—	−3.44	1.65	—	3.44		
1T′‐WSe_2_ + NO_2_	−5.03	−5.03	−5.03	0.00	5.07	—	0.14	1.5
2H‐WSe_2_ + NO_2_	−5.17	—	−3.53	1.64	—	3.53		

### 3.8 Gas Sensing Mechanism

**FIGURE 11:**
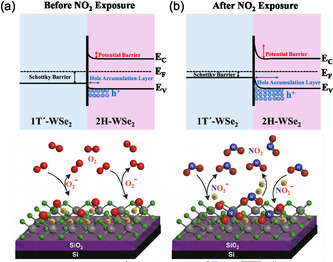
Schematic illustration of gas sensing mechanism of heterophase WSe_2_ via energy band diagram, (a) in ambient air and (b) when exposed to NO_2_.

The performance of the 2H‐1T′ heterophase WSe_2_ sensor is governed by a competitive adsorption model involving ambient oxygen and the target NO_2_ gas at room temperature. The overall response arises from weak physisorption of gas molecules on the semiconducting 2H basal planes together with more localized, stronger adsorption on the semi‐metallic 1T′ domains. A schematic representation of the proposed gas sensing mechanism of our heterophase WSe_2_ sensor is illustrated in Figure 11 for a deeper insight.

The influence of the ambient oxygen is critical, as the sensor was stabilized under dry synthetic air. Oxygen molecules from the ambient environment are readily adsorbed onto the surface of the WSe_2_ film (Equation (4)). While in situ spectroscopy is often utilized to observe these interactions directly, the fundamental gas‐surface dynamics can be firmly established by linking well‐accepted phenomenological thermodynamic models to our macroscopic electrical observations [102]. Owing to the high density of active sites at the 2H/1T′ phase boundaries and intrinsic reactivity of the semi‐metallic 1T′ phase, oxygen molecules are captured and extract electrons from the valence band of the material. As a result, surface‐adsorbed superoxide ions ($O_{2}^{-}$) are created, leading to the formation of a hole accumulation layer near the surface (Equation (5)), as schematically illustrated in Figure 11a. To understand the intrinsic contribution of the different phases to this response, we modeled the interaction of NO_2_ on the two phases of WSe_2_. Although the experimental baseline includes pre‐adsorbed oxygen, our DFT models target isolated NO_2_‐lattice interactions to identify the specific active sites driving the sensing signal. Insight into atomistic bonding mechanisms of NO_2_ on 2H and 1T′ domains of pristine WSe_2_ is important, and any additional effects arising from ambient oxygen as well as water molecules would be addressed by more complex simulations in future studies.

Upon exposure to NO_2_, gas molecules are adsorbed onto the basal planes of the semiconducting 2H‐WSe_2_. In this case, NO_2_ interacts through charge–transfer physisorption as it withdraws a small but measurable fraction of electrons from the valence band without strongly perturbing the electronic states of WSe_2_ (Equation (6)). This additional withdrawal of electrons increases the hole population in the 2H domain and thereby decreases the baseline resistance (Figure 11b). Although the net transferred charge per molecule is modest, it is sufficient to measurably modulate the valence band hole density and hence the conductance of the device. Based on our quantitative XPS analysis, the sensor surface is composed of an optimal ratio of approximately 83% 2H and 17% 1T′ phases. This specific phase distribution is fundamentally critical to the synergistic transduction mechanism. If the film were entirely 1T′, its metallic nature would prevent the macroscopic modulation of a semiconducting baseline, thereby quenching the chemiresistive response. Conversely, a pure 2H film suffers from sluggish kinetics due to higher activation energies for gas adsorption. In our heterophase architecture, the dominant 2H matrix (∼83%) provides the necessary semiconducting baseline and continuous hole‐percolation network required for measurable resistance changes. Simultaneously, the ∼17% fraction of 1T′ domains provides a concentrated density of high‐energy catalytic anchors. Our calculations reveal that NO_2_ adsorption on the 1T′ phase is approximately 52% stronger than on the 2H phase with a significantly shorter adsorption distance. This transition toward weak chemisorption, facilitated by orbital hybridization between NO_2_ and the reactive Se atoms, effectively traps the analytes. In addition to direct adsorption, the sensing response is amplified by the displacement of pre‐adsorbed oxygen. Because NO_2_ binds more strongly to the surface than O_2_, it competitively displaces the adsorbed oxygen ions and releases neutral oxygen back into the gas phase while retaining the captured negative charge on the surface (Equation (7)). Furthermore, the electronic modulation at the 2H‐1T′ interface plays a critical role in the signal transduction [103]. The cumulative electron withdrawal by both the physisorbed NO_2_ (on 2H WSe_2_) and chemically adhered NO_2_ (on 1T′ WSe_2_) lowers the Fermi level. This electronic modulation facilitates hole transport through the percolating network, amplifying the resistive response. In future work, transport calculations for key adsorption configurations will be carried out to clarify the origins of the 40% sensor response.

Upon removal of the NO_2_ supply, the sensor resistance recovered to its baseline. Although DFT calculations suggest a mechanism dominated by physisorption (which typically implies rapid desorption), the experimental recovery time was observed to be approximately 40 min. This discrepancy can be attributed to diffusion‐limited kinetics rather than strong chemical bonding [104], but could also be due to the lack of oxygen effects in simulations. While the intrinsic adsorbate–substrate bond is weak, the macroscopic recovery is governed by the extrinsic morphology of the heterophase film. NO_2_ molecules trapped within the porous network of the stacked 2H‐1T′ layers likely undergo multiple re‐adsorption/desorption events before fully diffusing out of the sensing layer. Additionally, the higher binding energy sites at the phase boundaries likely act as shallow traps, prolonging the desorption process at room temperature.

(4) $$O_{2}\left(g\right)\to O_{2}(\mathrm{ads})$$

(5) $$O_{2}\left(\mathrm{ads}\right)+e^{-}\to O_{2}^{-}(\mathrm{ads})$$

(6) $${\mathrm{NO}}_{2}(g)+\delta e^{-}\to {\mathrm{NO}}_{2}^{-\delta }(\mathrm{ads})$$

(7) $${\mathrm{NO}}_{2}(g)+O_{2}^{-}(\mathrm{ads})\to {\mathrm{NO}}_{2}^{\delta -}(\mathrm{ads})+O_{2}(g)$$

## 4 Conclusions

In this work, we have demonstrated a hydrogen‐free high‐yield synthesis of heterophase WSe_2_ for ultralow‐level detection of NO_2_ with high precision. The as‐synthesized WSe_2_ exhibited a distinctive nano‐butterfly morphology comprising highly crystalline, vertically oriented nano‐triangular arrays that effectively prevent restacking and maximize gas diffusion pathways. Crucially, the successful integration of the 2H/1T′ heterophase overcame the kinetic limitations of conventional TMDs by coupling semiconducting stability with metallic active sites. Notably, the synthesis strategy is scalable and sustainable, facilitating mass production at an industrial scale. The sensor exhibited a very high response of 40% toward 800 ppb at room temperature, indicating high sensitivity toward trace levels of NO_2_. Besides, the device demonstrated unparalleled selectivity against interfering reducing gases, ensuring high‐fidelity sensing. In addition, the sensor exhibited remarkable long‐term stability and reproducibility, maintaining consistent performance over an extended period of 40 weeks. DFT‐based computational results validated the experimental findings, establishing that the coupling between the NO_2_ absorbate and the WSe_2_ substrate is predominantly physisorptive in nature. To further validate the proposed sensing mechanism at the molecular level, future research will prioritize direct in situ spectroscopic techniques. Employing operando ambient‐pressure XPS or DRIFTS would offer real‐time insights into surface adsorbed species and the dynamics of gas–surface interactions. Moreover, the sensor displayed an enhanced response across several humidity levels for room temperature sensing, endorsing its applicability in real‐world sensing environments. Therefore, this work paves the way for advancing TMD‐based gas sensing technologies, offering transformative potential for environmental monitoring.

## Funding

This study was supported by Horizon 2020 research and innovation program (Grant 945413), Agencia Nacional de Investigación e Innovación (Grant PID2022‐142451OB‐C21), Ramon iy cajal program (Grant RYC2022‐038111‐I), Institució Catalana de Recerca i Estudis Avançats, and Research council of finland (Grant 352727).

## Conflicts of Interest

The authors declare no conflicts of interest.

## Supporting information

Supplementary Material

## Data Availability

The data that support the findings of this study are available from the corresponding author upon reasonable request.
