# Supplementary material for: Hydrogen‐Free APCVD Synthesis of Heterophase WSe2 Nano‐Butterflies for Room Temperature NO2 Detection: Experimental and Computational Insights
Source: Small Sci. 2026 Jun 8;6(6):e70322. doi: 10.1002/smsc.70322 (PMC13248848; doi:10.1002/smsc.70322)
Supplement: Supplementary file 1 — Supplementary Material [file SMSC-6-e70322-s001.pdf]

Supplementary Information

Hydrogen-Free APCVD Synthesis of Heterophase WSe_2 Nano-butterflies for Room Temperature NO_2 Detection: Experimental and Computational Insights

Mubdiul Islam Rizu [1,2,3], Abhilash Patra [4], Fatima Ezahra Annanouch [1,2,3], Milica Todorović [4]*, Dalal Fadil [1,2,3], Eduard Llobet [1,2,3]*

1. Universitat Rovira i Virgili, MINOS, School of Engineering, Avda. Països Catalans 26, 43007 Tarragona, Spain.
2. IU-RESCAT, Research Institute in Sustainability, Climatic Change and Energy Transition, Universitat Rovira i Virgili, Joanot Martorell 15, 43480 Vila-seca, Spain.
3. TecnATox - Centre for Environmental, Food and Toxicological Technology, Universitat Rovira i Virgili, Avda. Països Catalans 26, 43007 Tarragona, Spain.
4. Department of Mechanical and Materials Engineering, Faculty of Technology, University of Turku, 20500 Turku, Finland.

*Corresponding Author: fatimaezahra.annanouch@urv.cat, milica.todorovic@utu.fi

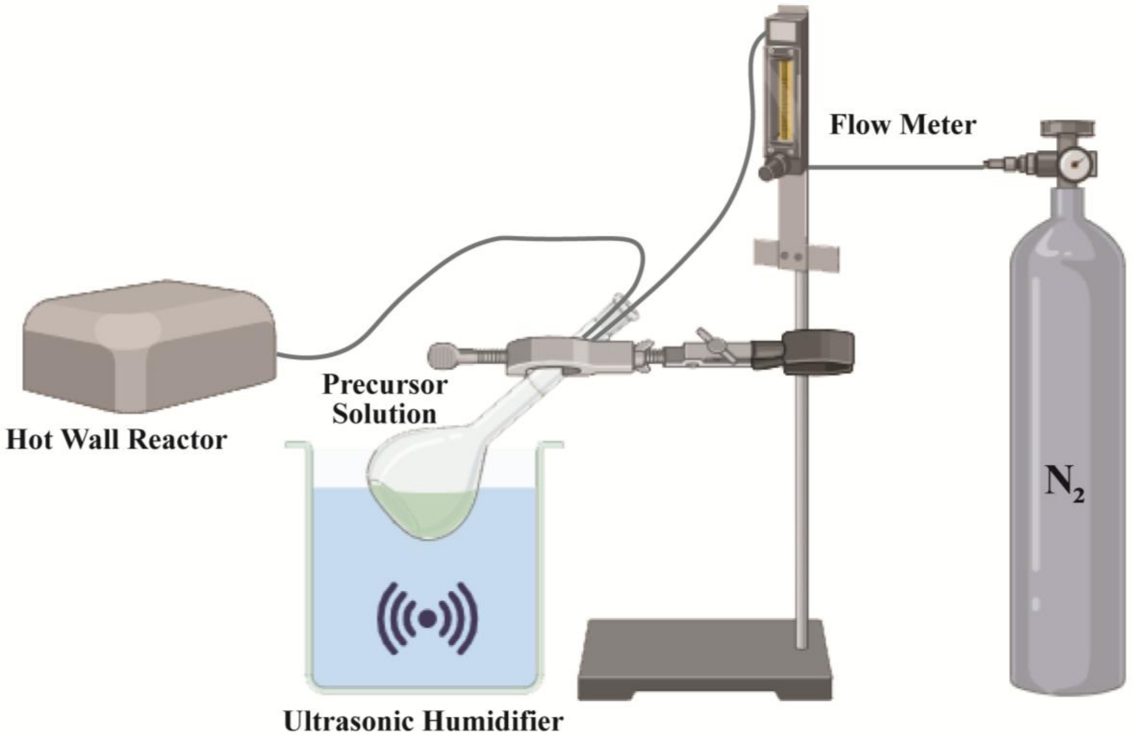

Fig. S1. Schematic image of the deposition of WO_3 film by AACVD.

Parameters	FESEM Micrographs	Raman Spectroscopic Analysis
Temperature<br>850 °C<br>Dwell Time<br>1 hour	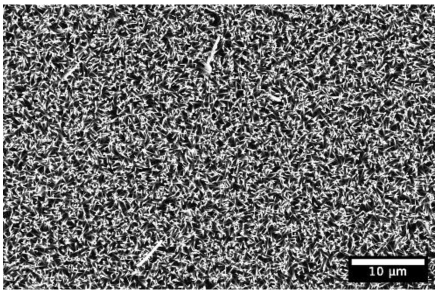	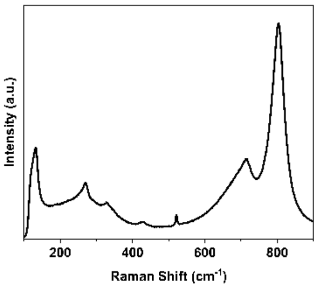
Temperature<br>900 °C<br>Dwell Time<br>1 hour	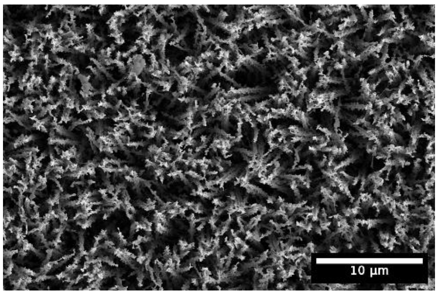	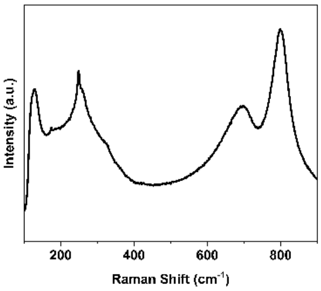
Temperature<br>950 °C<br>Dwell Time<br>1 hour	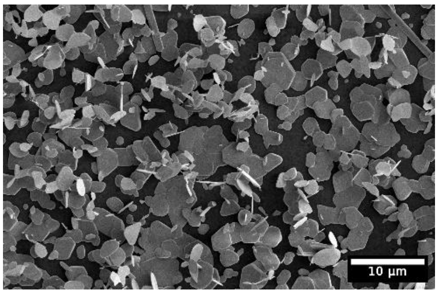	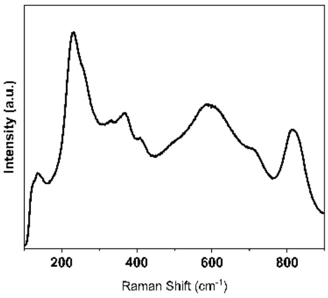
Temperature<br>900 °C<br>Dwell Time<br>0.5 hour	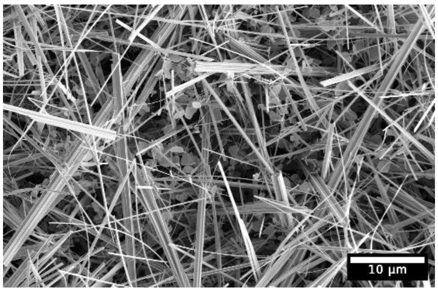	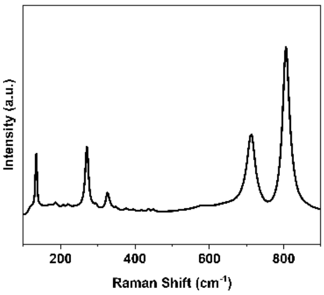
Temperature<br>900 °C<br>Dwell Time<br>1.5 hour	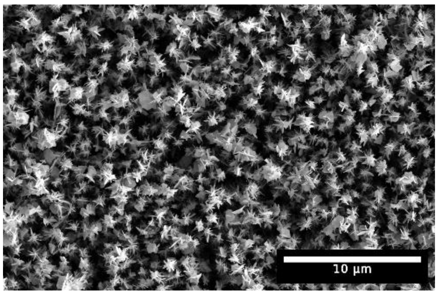	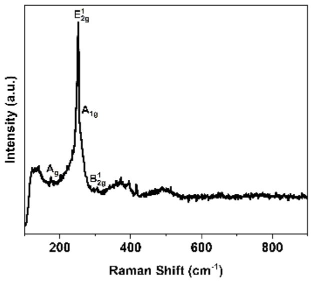

Fig S2. Morphological and structural evolution of the WSe_2 films under varying temperature and temporal gradients.

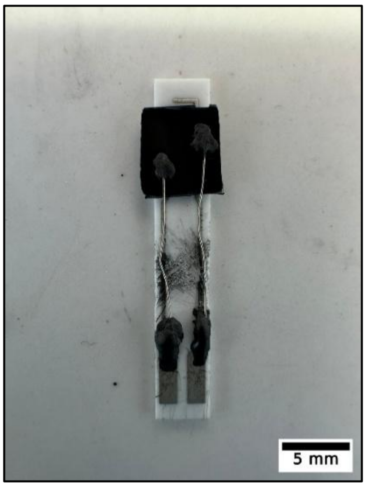

Fig. S3. Optical photograph of the fabricated WSe_2 sensor device.

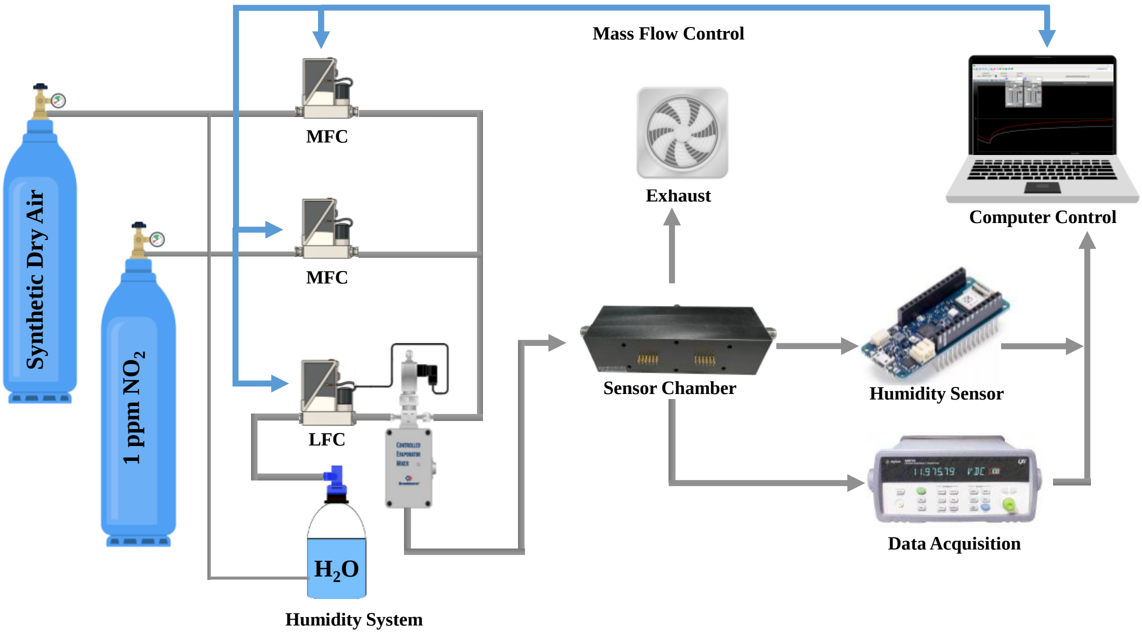

Fig. S4. Schematic representation of customized gas measurement system.

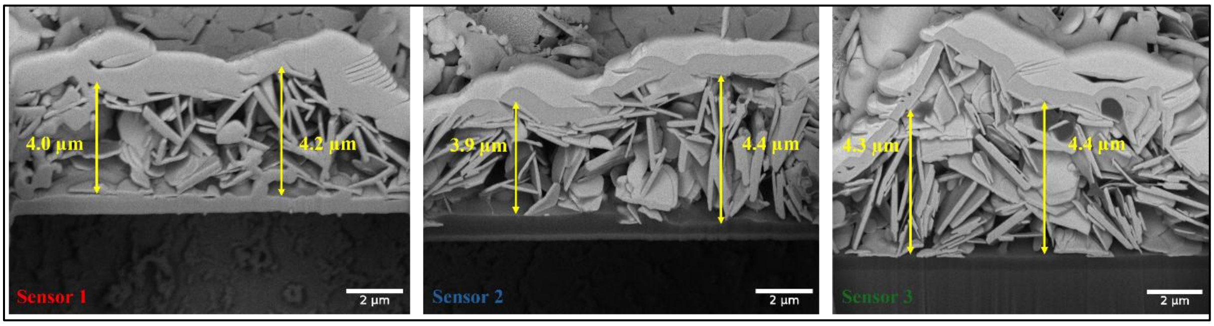

Fig. S5. Cross-sectional thickness analysis across three sensing devices.

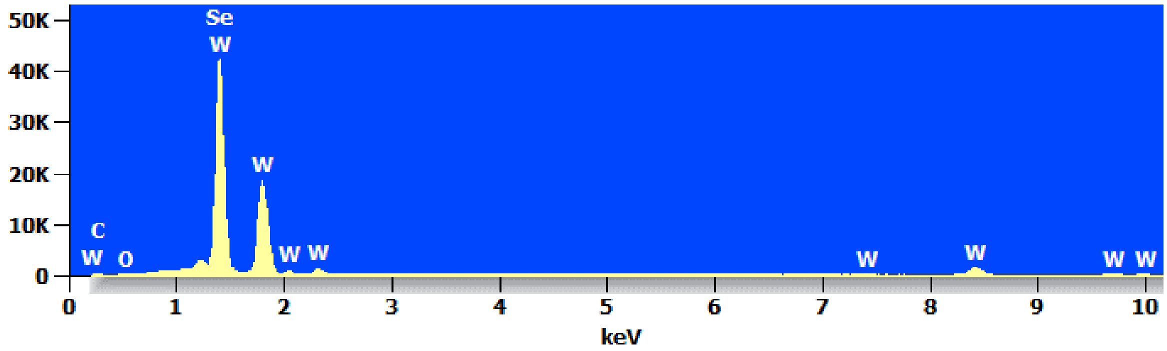

Fig. S6. EDX analysis of as-grown WSe_2 revealing the strong presence of W and Se.

Table S1. Elemental Analysis

Element	Weight (%)	Atom (%)
**C**	2.21	17.32
**O**	0.35	2.07
**Se**	45.03	53.74
**W**	52.41	26.86
**Total**	100	100

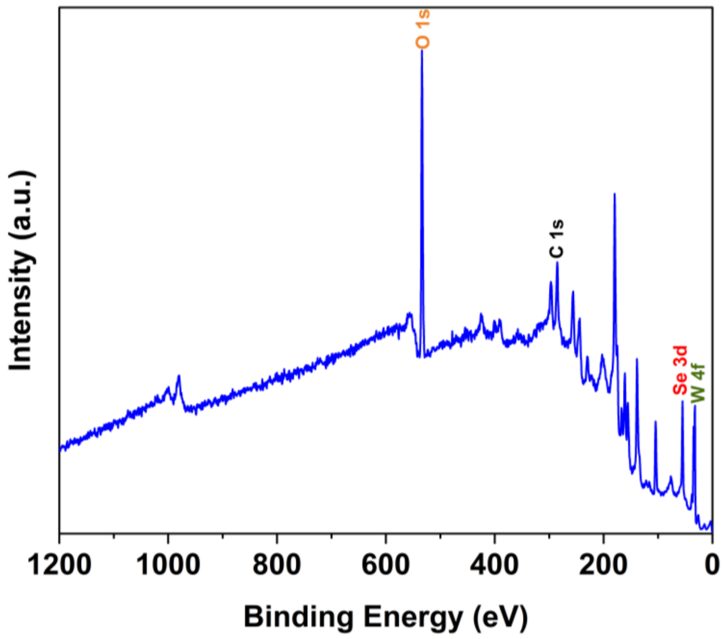

Fig. S7. XPS survey spectrum of as-grown WSe_2.

Table S2. Percentage of the corresponding areas of distinct phases of as-synthesized WSe_2

	**W 4f Core Level**		**Se 3d Core Level**	
**Phase**	2H	1T′	2H	1T′
**% Area Fraction**	84.49	15.51	81.08	18.92

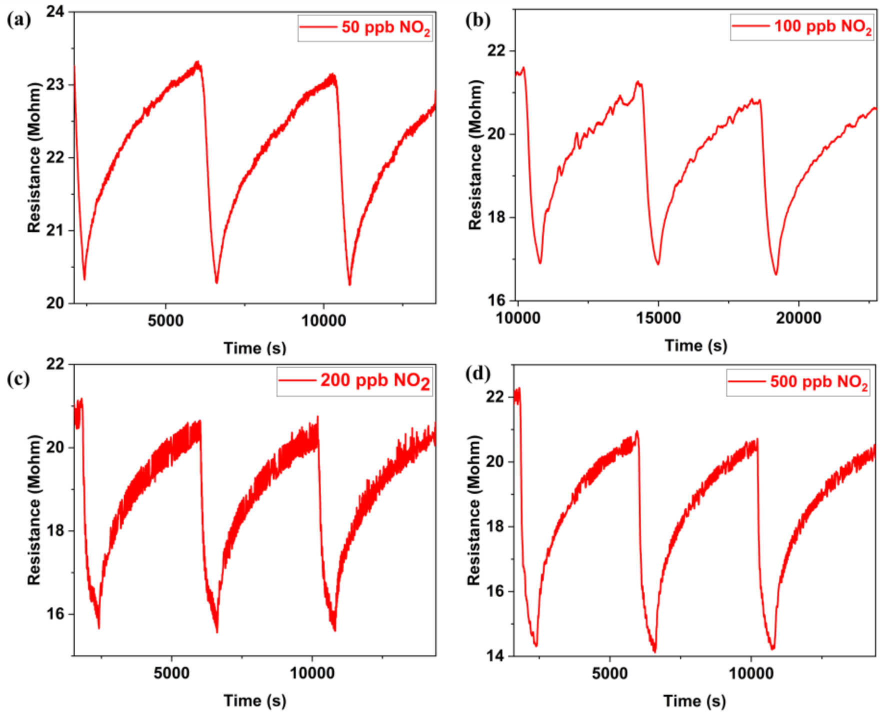

Fig. S8. Sensing repeatability in dry condition for (a) 50 ppb, (b) 100 ppb, (c) 200 ppb and (d) 500 ppb NO_2.

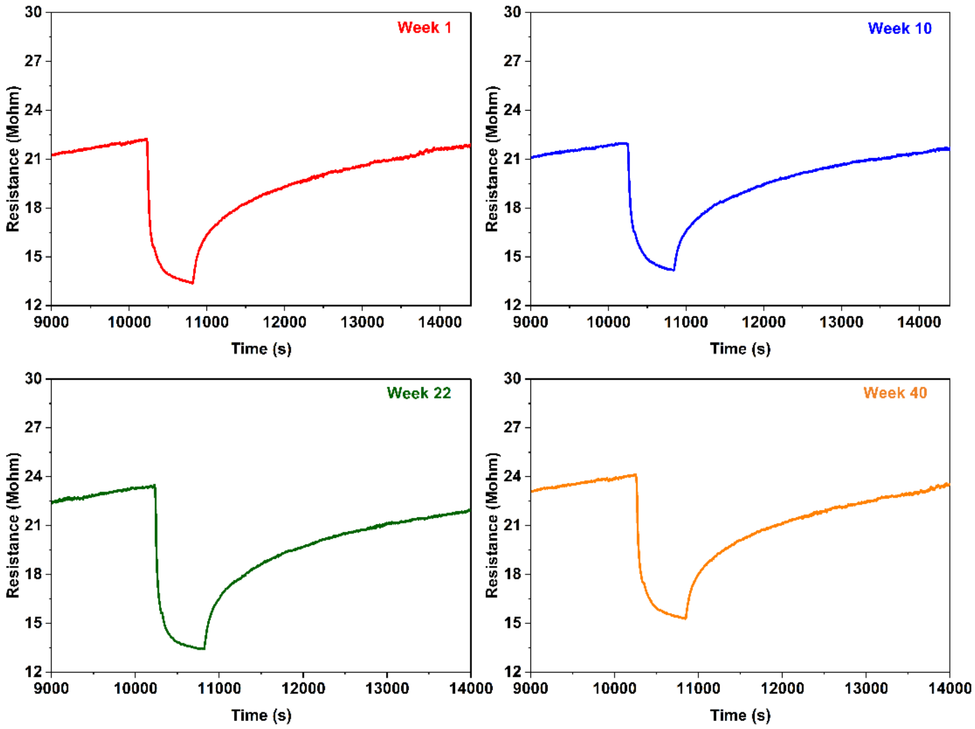

Fig. S9. Comparative resistance transients over 40 weeks.

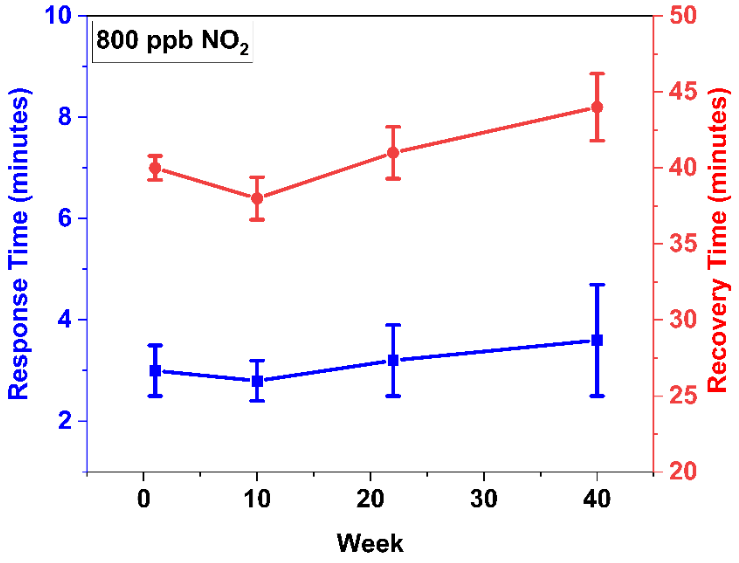

Fig. S10. Response and recovery behaviour over 40 weeks. Error bars represent the mean ± standard deviation derived from n = 3 independent sensors.

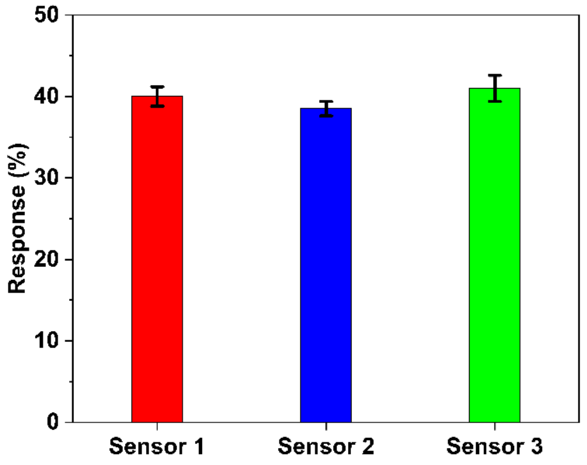

Fig. S11. Device-to-device reproducibility test. Error bars represent the mean ± standard deviation derived from n = 3 independent measurements.

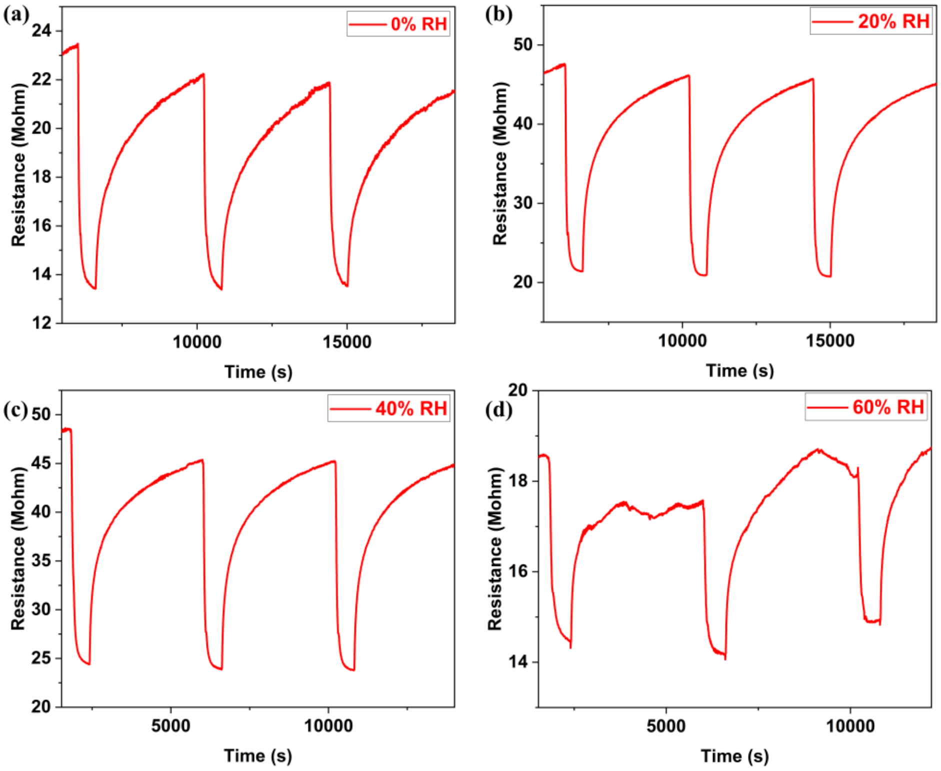

Fig. S12. Sensing repeatability of 800 ppb NO_2 in humid condition for (a) 0% RH, (b) 20% RH, (c) 40% RH and (d) 60% RH.
